# Gummy Stem Blight Resistance in Melon: Inheritance Pattern and Development of Molecular Markers

**DOI:** 10.3390/ijms19102914

**Published:** 2018-09-25

**Authors:** Md Zahid Hassan, Md Abdur Rahim, Sathishkumar Natarajan, Arif Hasan Khan Robin, Hoy-Taek Kim, Jong-In Park, Ill-Sup Nou

**Affiliations:** Department of Horticulture, Sunchon National University, 255 Jungang-ro, Suncheon, Jeonnam 57922, Korea; zhassan.pstu@gmail.com (M.Z.H.); rahimgepb@scnu.ac.kr (M.A.R.); sathisbioinfo@gmail.com (S.N.); gpb21bau@gmail.com (A.H.K.R.); htkim@sunchon.ac.kr (H.-T.K.); jipark@sunchon.ac.kr (J.-I.P.)

**Keywords:** inheritance, GSB, NBS-LRR, InDel, phenotype, genotype

## Abstract

Gummy stem blight (GSB) causes enormous losses to melon (*Cucumis melo* L.) production worldwide. We aimed to develop useful molecular markers linked to GSB resistance. In this study, 168 F_2_ plants were obtained from the F_1_ population of a cross between the GSB-susceptible ‘Cornell ZPPM 339’ and the GSB-resistant ‘PI482399’ lines. A 3:1 ratio of susceptible and resistant genotypes was observed in the F_2_ population, indicating control by a single recessive gene. Nucleotide-binding site leucine-rich repeat (NBS-LRR) genes confer resistance against insects and diseases in cucurbits including melon. We cloned and sequenced the TIR-NBS-LRR-type resistance gene MELO3C022157, located on melon chromosome 9, from resistant and susceptible lines. Sequence analysis revealed deletions in the first intron, a 2-bp frameshift deletion from the second exon and a 7-bp insertion in the 4th exon of the resistant line. We developed two insertion/deletion (InDel) markers, GSB9-kh-1 and GSB9-kh-2, which were found in the first intron of MELO3C022157 linked to GSB resistance. We validated these markers with the F_2_ population and inbred lines. These InDels may be used to facilitate marker-assisted selection of GSB resistance in melon. However, functional analysis of overexpressing and/or knock-down mutants is needed to confirm the frameshift mutation.

## 1. Introduction

Cultivated melon (*Cucumis melo* L.) is an economically important member of the Cucurbitaceae family, which includes a diverse group of annual trailing vine plants, such as honeydew, cantaloupe, mango melon, snake melon, snap melon, and pickling melon [1]. Consumer preference for melon variety is determined mostly by sweetness, aroma, flavor, texture, and the presence of health-promoting vitamins and minerals [2,3]. However, pathogen attack is a major barrier to achieving higher yields of this important fruit crop.

Gummy stem blight (GSB), which is caused by the fungal pathogen *Didymella bryoniae* (Auersw.) Rehm, is a devastating disease of cucurbitaceous crops, including melon, throughout the world [4,5]. Disease symptoms can appear on all parts of the plant (e.g., leaves, stems, and fruits), except the roots. Early symptoms of the disease include yellowing of the leaf margins (chlorosis), while at later stages, this yellowing spreads to form light to dark brown spots (necrosis) across the entire leaf surface. This results in stem canker of the cortical tissue that produces a characteristic brown, gummy exudate that is caused by water soaking of the leaves and hypocotyls. Consequently, in a susceptible interaction, these lesions linger and expand, ultimately girdling the stem and leading to wilting and plant death [6].

Limited success has been achieved in controlling GSB with chemical methods; therefore, the most effective means of GSB management is to use resistant cultivars [5,7]. The agricultural importance of this disease means that the search for genetic resistance has been ongoing for some time, yet, to date, most of the available GSB-resistant melon cultivars and breeding cultivars come from one line, plant introduction 140471 (‘PI140471’) [1,8]. However, ‘PI140471’ has failed to achieve satisfactory resistance [6,9]. 

In the search for further resistant lines, genetic analyses have explored several independent GSB resistance loci from diverse cultigens of melon. These include five independent, single genes conferring resistance to GSB, four of which are dominant and one of which is recessive [10,11,12]. Recently, eight GSB resistance genes have been reported, all being located on chromosome 4 [13]. Molecular markers that are associated with melon GSB remain limited, and most of those reported are either amplified fragment length polymorphisms (AFLP), or simple sequence repeats (SSR) [7,14]. Presently, insertion/deletion (InDel) markers are recognized as an effective marker system to complement other sequence-based genetic markers such as SSRs and single nucleotide polymorphisms (SNPs). This is mainly because these markers include myriad desirable, inherent genetic features, including co-dominant and multi-allelic inheritance and wide genomic distribution [15]. The lack of effective molecular markers that are linked to GSB resistance hampers the transfer of resistance loci to commercial melon cultivars. The development of molecular markers linked to GSB resistance is key to be able to detect and pyramid multiple desirable alleles in a collection of different melon genotypes. 

Comparative genomic analysis revealed that chromosome 5 of cucumber has a syntenic relationship with chromosome 9 of melon [16,17]. A stable quantitative trait locus (QTL; *gsb5.1*) for GSB resistance was also detected on chromosome 5 in cucumber [18]. Moreover, the second largest cluster of nucleotide-binding site leucine-rich repeat (NBS-LRR) genes has been reported on melon chromosome 9 [19]. Of these NBS-LRR genes, *Fom1* and *Prv* confer resistance against *Fusarium oxysporum* and Papaya ring-spot virus, respectively. Therefore, we focused our efforts on melon chromosome 9 to look for candidate GSB resistance genes.

In the plant genome, the NBS-LRR genes are often found to be resistance (*R*) genes, and they often occur in clusters at specific loci [20]. NBS-LRR-type *R* genes are vital in plant responses to various pathogens, including viruses, bacteria, fungi, and nematodes [21]. *R* genes have also been reported in several other plant species, including *Arabidopsis*, rice, and cucumber [21,22,23].

To date, our research has been limited in terms of identifying molecular markers that are associated with GSB in melon. In the present study, we redoubled our efforts to analyze the inheritance patterns and development of molecular markers associated with GSB resistance in melon.

## 2. Results

### 2.1. Inheritance of GSB Resistance in Melon

**Figure 1:**
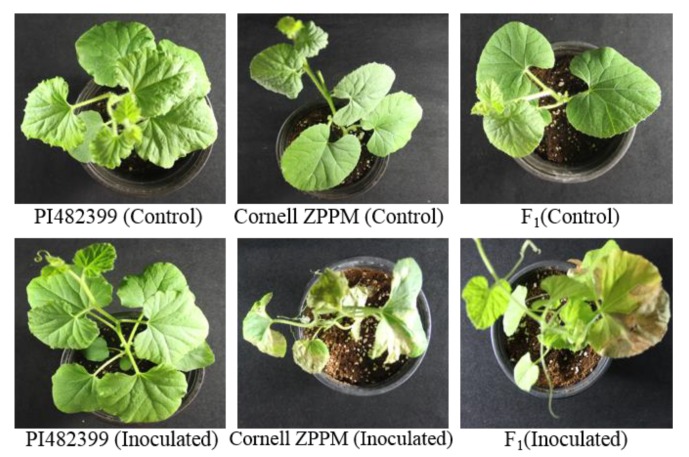
Phenotypes of the two melon lines, ‘PI482399’ (resistant) and ‘Cornell ZPPM 339’ (susceptible), and their F_1_ (susceptible) generation 14 days after being inoculated with *Didymella bryoniae*.

The inheritance pattern of GSB resistance was analyzed in the F_2_ population of melon. Bioassay results revealed that the F_1_ plants that were derived from a cross between ‘Cornell ZPPM 339’ and ‘PI482399’ were disease-susceptible, suggestive of a recessive trait (Figure 1). A total of 168 F_2_ plants were analyzed, including 42 resistant and 126 susceptible plants. A Chi-square test showed that GSB resistance segregated in a ratio of 3:1 (susceptible:resistant), which is consistent with a monogenic recessive trait (Table 1 and Figure S1).

### 2.2. Selection of GSB Resistance Genes

**Figure 2:**
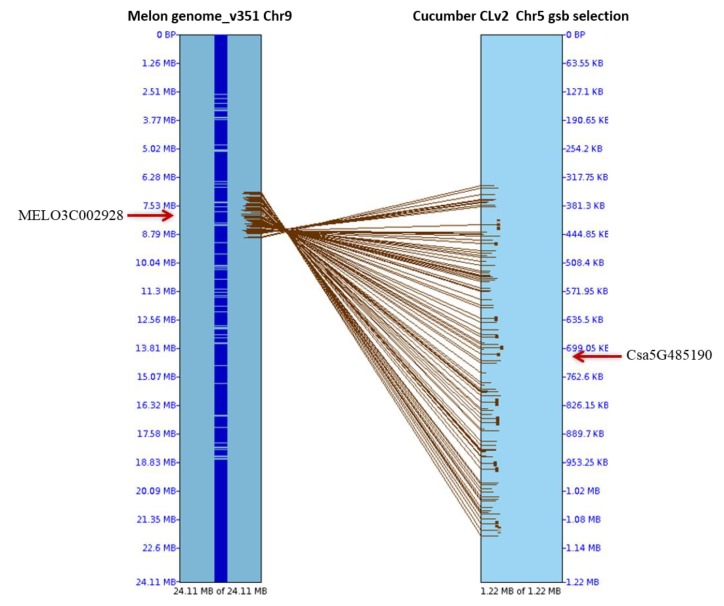
Alignment of cucumber gummy stem blight-linked quantitative trait locus (QTL) areas (*GSB5.1* in chromosome 5 [18]) with the genome of melon chromosome 9 using SyMAP v3.4. The resistant melon gene MELO3C002928 in chromosome 9 has synteny with the cucumber *R* gene CsaG485190 associated with GSB resistance on chromosome 5.

**Figure 3:**
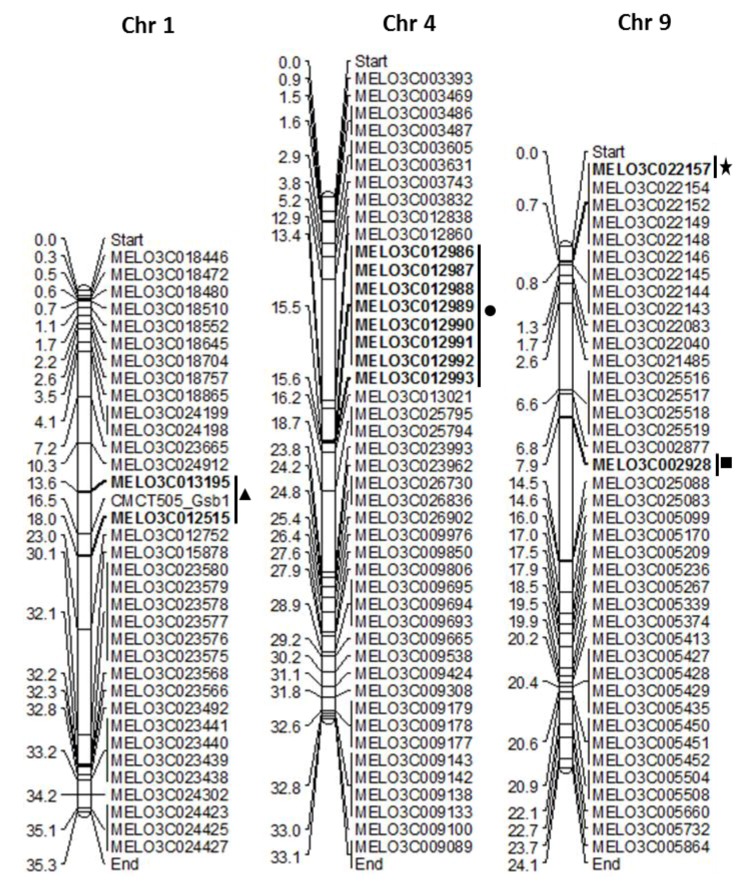
Construction of chromosomal map chart of resistance (*R*) genes from melon chromosomes 1, 4 and 9. The solid triangle indicates closest *R* genes to a previously reported simple sequence repeats (SSR) marker for resistance to gummy stem blight, CMCT505_GSB1 in chromosome 1 [14]; solid circle represents reported GSB resistance genes in chromosome 4 [13]; the asterisk indicates polymorphic TAIR-NBS-LRR gene resistance to GSB in chromosome 9; solid rectangle indicates melon *R* gene MELO3C002928 has synteny with cucumber *R* gene Csa5G485190, which is linked to gummy stem blight resistance QTL *GSB5.1* in cucumber chromosome 5 [18].

**Figure 4:**
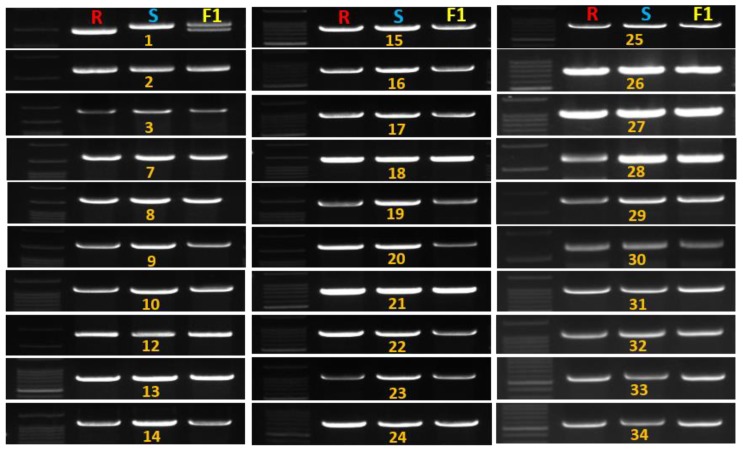
Banding profile of melon resistance genes with the polymorphic nucleotide-binding site leucine-rich repeat (NBS-LRR) gene MELOC022157 (1). R indicates resistant line (‘PI482399’), S indicates susceptible line (‘Cornell ZPPM 339’) and F_1_ (S × R). Serial 1–3, 7–34 indicates amplified genes, and absent serial number 4–6, 35 are not amplified (genes list in Table 2).

The identification of GSB-resistant genotypes is essential to develop GSB-resistant melon cultivars. However, PCR amplification detected no polymorphism to closely related resistance genes (the nearest *R* genes of GSB-linked SSR marker and eight previously reported GSB *R* genes) (Figure S2). After aligning the cucumber GSB-linked QTL areas, 114 genes were found in melon chromosome 9, of which only one was an *R* gene (MELO3C002928) based on its the synteny with the cucumber *R* gene Csa5G485190 [18], which is known to be associated with GSB resistance (Figure 2 and Figure 3, Table S1). Among 35 *R* genes on melon chromosome 9, 30 were amplified, including the previously reported *R* gene MELO3C002928 [24]. Only one gene, MELO3C022157, encoding a TIR-NBS-LRR domain, showed high polymorphism between the resistant and susceptible lines in the F_1_ population (Figure 4).

### 2.3. Cloning and Sequencing of GSB Candidate Gene

**Figure 5:**
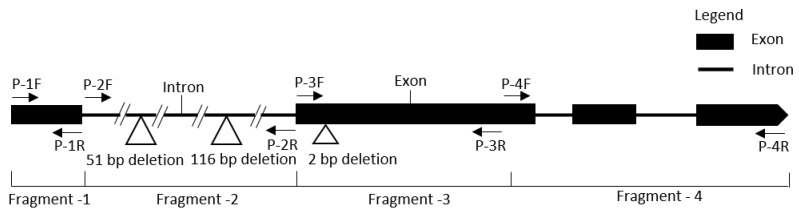
Exon–intron distribution of the NBS-LRR gene MELO3C022157 shows primers in different locations.

**Figure 6:**
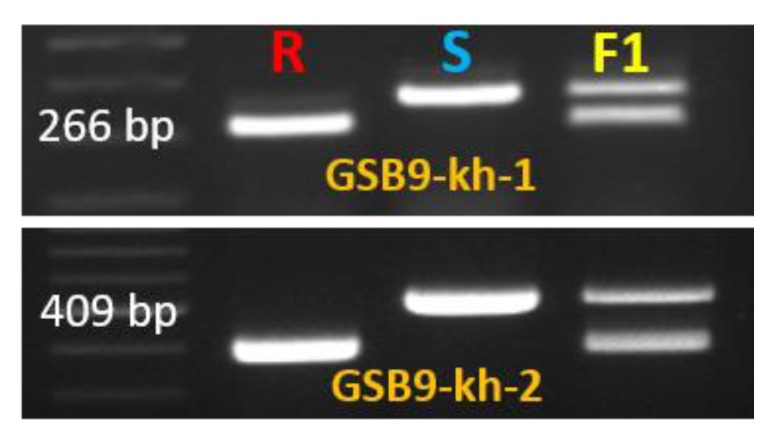
Polymorphic InDel markers Gsb9-kh-1 and Gsb9-kh-2 linked to gummy stem blight in melon. R indicates resistant line (‘PI482399’), S indicates susceptible line (‘Cornell ZPPM 339’) and F1 indicates ‘Cornell ZPPM 339’ × ‘PI482399’.

To discover the specific polymorphic region responsible for GSB resistance, primers were designed to anneal to four different locations of the candidate *R* gene MELO3C022157, which showed polymorphism between resistant and susceptible lines (Figure 5 and Table S2). After PCR amplification, the polymorphic region was identified in the first intron of the gene (Figure 6 and Figure S3). Furthermore, upon cloning and sequencing, the candidate gene MELO3C022157, several InDels and SNPs were identified in both the intron and exon regions between resistant and susceptible lines (Figure S4A). In addition, there was a 2-bp deletion at the 331 nt position of the second exon of the resistant line ‘PI482399’, which generates a premature stop codon (TGA) that causes a frameshift mutation and produces a truncated protein (Figure S4B,C). Moreover, a 7-bp insertion was detected at the 1956 nt position of the 4th exon of the resistant line (Figure S4C).

### 2.4. InDel Detection and Marker Development

Sequence analysis revealed two long deletions (51 and 116 bp in length) in the first intron of the resistant line ‘PI482399’ when compared to the susceptible line (Figure 5 and Figure S4A). Therefore, primers were designed separately to cover two InDel regions. PCR amplification using these primers also confirmed the existence of InDels GSB9-kh-1 (266 bp) and GSB9-kh-2 (409 bp), which were able to perfectly distinguish resistant and susceptible lines (Figure 6 and Table 3).

### 2.5. Validation of the InDel Marker

**Figure 7:**
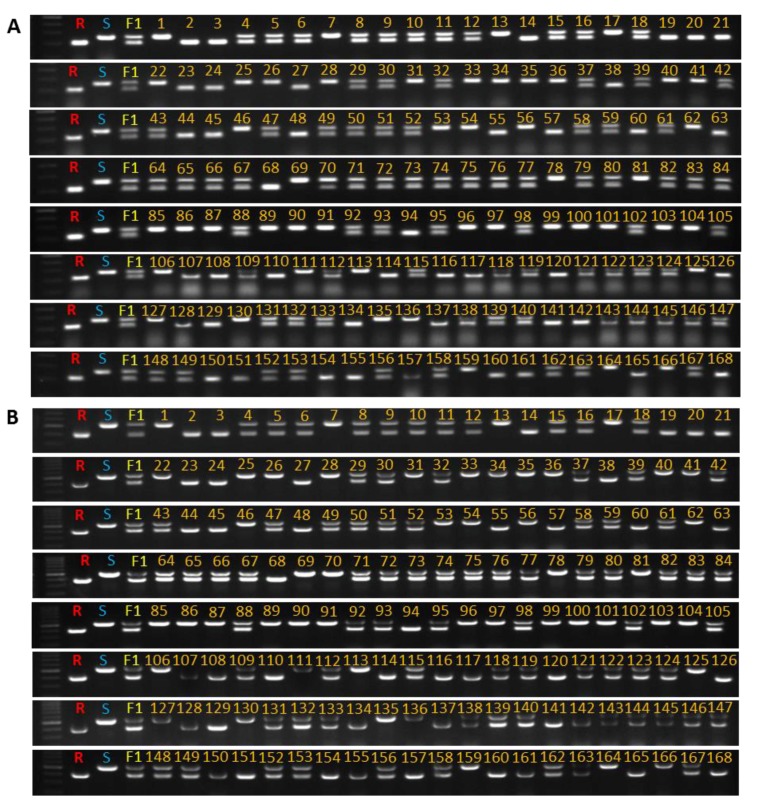
Validation of InDel marker GSB9-kh-1 (**A**) and GSB9-kh-2 (**B**) in the F_2_ population (1–168) of melon. R indicates resistant line (‘PI482399’), S indicates susceptible line (‘Cornell ZPPM 339’), and F_1_ indicates (‘Cornell ZPPM 339’ × ‘PI482399’).

Two populations were chosen to validate the InDel markers GSB9-kh-1 and GSB9-kh-2. The first comprised 168 melon plants from the F_2_ population, and the second contained 15 inbred melon lines. In the case of the F_2_ population, molecular assay results supported the bioassay-based phenotypic results (Figure 7 and Table S3). Of the 15 inbred lines, three ‘PI482398’, ‘PI353814’, and ‘PI504558’ were shown to be resistant (Figures S5 and S6, and Table S4). In both cases, genotypic results that were obtained using the InDel markers matched the phenotypic expectations perfectly.

## 3. Discussion

The ultimate goal of melon breeders is to increase yield. However, GSB infections often cause severe yield loss. Although chemical control, such as application of fungicides, has had great success, the repeated use of fungicide is not advisable as a long-term solution because of the negative effects of pesticides on the environment [7]. Therefore, marker-assisted selection (MAS) has been effectively used as an alternative to phenotypic selection in attempts to breed disease-resistant cultivars.

Plants have developed sophisticated sensitivity and response mechanisms that interpret pathogen signals and induce proper defenses. The best-studied defense system is mediated by a specific interaction between the products of a single *avirulence* (*Avr*) gene from the pathogen, and a single *R* gene from the plant. This is also called the ‘gene-for-gene’ model [25]. The results that were obtained from phenotyping the F_1_ and F_2_ melon populations and their parents confirm the Mendelian segregation ratio of 3:1 (susceptible:resistant to GSB), with a recessive gene that confers resistance to disease. In this way, it can be substantiated that the inheritance of resistance in our source plants is monogenic and recessive, because all plants in the F_1_ population were completely susceptible to GSB. Similar result was also reported for GSB resistance in melon [12]. 

In recent years, comparative genomics research has seen rapid development with the release of the cucumber and melon genomes. In plant genomes, the NBS-LRR class of genes, which often occur in clusters at specific loci, include groups of resistance genes [20]. In plants, TIR-NBS-LRR-type *R* genes are known to be involved in resistance against diverse pathogens, including fungi, bacteria, viruses, nematodes, oomycetes, and insects [26,27]. On the contrary, Lorang et al. [28] have been reported a disease susceptibility gene *LOV1*, which belongs to the NBS-LRR *R* gene family.

In our study, we found the melon *R* gene MELO3C002928, located on chromosome 9, to be amplified in both resistant and susceptible lines. This was identified by aligning the cucumber gene harbored at the *gsb5.1* QTL region to the melon genome, and indicates that chromosome 9 might be linked to GSB resistance in melon. Accordingly, the polymorphism of the *R* gene MELO3C022157 between resistant and susceptible lines signifies that MELO3C022157 might be associated with GSB resistance in melon. Further, BLAST searches of the MELO3C022157 gene against the NCBI, TAIR10, TrEMBL and Swiss-Prot databases revealed that this *R* gene has similarity with a Tobacco Mosaic Virus (TMV) resistance protein and a FOM-1 (*Fusarium oxysporum*) resistance protein in melon (Table S5). Similarity was also found to *Fom-2*, an NBS-LRR-type gene that is involved in resistance against *Fusarium* wilt in melon, and *pmr2*, an NBS-LRR class *R* protein, which confers resistance to powdery mildew in watermelon [29,30]. In some other plant species, it has also been reported that the NBS-encoding *R* genes confer resistance against different plant pathogens. For example, *Bol037156* (*FOC1*) [31] is known to confer resistance against *Fusarium* wilt in *Brassica oleracea*, and RPS6 [32] is involved in resistance against *Pseudomonas syringae* in *Arabidopsis*. Furthermore, RCY1, an LRR class *R* protein, confers resistance to Cucumber Mosaic Virus in *Arabidopsis* [29,33]. A recent study of NBS-encoding *R* genes in heading cabbage found that expression of some candidate *R* genes was significantly higher in black rot-resistant lines when compared to susceptible lines [34].

At present, intron length polymorphism (ILP) is considered to be a valuable source for developing genetic markers with high interspecies transferability [35,36]. ILP markers in resistance gene analogs have been successfully utilized in different crops, including maize [36] and sunflower [37].

Cloning and sequencing the MELO3C022157 gene from resistant and susceptible lines revealed two long deletions in the first intron region of the resistant line, indicating that mutations may have occurred in the resistant line during evolution. Moreover, 2-bp deletions in the second exon may be the result of a frameshift mutation in the resistant line (Figure S4C).

Our results suggest that InDels that cause frameshift mutations may have yielded functional evolutionary intermediates, and might be an effective means of sequence divergence. In turn, this might be the cause of resistance in the melon line ‘PI482399’. However, functional analysis of the MELO3C022157 gene is needed to test this hypothesis. Recent reports have shown that frameshift-inducing InDels were frequently bypassed to give functional proteins at surprisingly high frequencies [38]. 

InDels can lead to an abundance of genetic markers that are widely spread across the genome. It has also been reported that, in some crops, InDel markers are more polymorphic than SSRs [39,40]. However, to date, no research has been undertaken to explore the role of InDel markers for GSB resistance in melon. In this study, the newly developed, co-dominant InDel markers GSB9-kh-1 and GSB9-kh-2, were able to perfectly differentiate between homozygous and heterozygous genotypes in the F_2_ population, which followed the Mendelian 1:2:1 ratio with a phenotypic agreement of 100%. However, the SSR marker CMCT505, which has been linked to *Gsb1* adaptability, had a phenotypic agreement of 83% (Figure S7, Tables S6 and S7). The identical results of InDel marker assays and phenotypic assays in the melon lines ‘PI482398’, ‘PI353814’ and ‘PI504558’, along with the control line ‘PI482399’, suggest that these lines might contain a GSB resistance gene on chromosome 9.

To date, PCR-based co-dominant DNA markers are the most convenient, reliable, and cost-effective molecular markers available to use in practical breeding programs using MAS [41]. To our knowledge, this is the first report of InDel marker development for GSB resistance in melon.

## 4. Materials and Methods

### 4.1. Plant Materials

The male parental line that was used in this study was ‘PI482399’, which is highly resistant to GSB. The female parental line was ‘Cornell ZPPM 339’, which is highly susceptible to GSB. Both were collected from the Department of Plant Breeding and Genetics, Cornell University, Ithaca, NY, USA (Figure 1). ‘PI482399’ was crossed with ‘Cornell ZPPM 339’ to develop the F_1_ generation. The resistance and susceptibility of these lines were also confirmed with bioassay by Frantz and Jahn [12]. The seeds were sown in a commercial nursery soil mixture at plant growth chamber. After three weeks, plants were transferred to the glasshouse.

### 4.2. Development of F_2_ Population to Explore Inheritance Patterns

The F_2_ generation of 168 plants was developed via self-pollination of the F_1_ population. As well as this F_2_ population, 15 melon inbred lines were also used to validate the markers (Table S8). All of the plants were grown in the glasshouse at the Department of Horticulture, Sunchon National University, South Korea.

### 4.3. Fungal Isolate and Inoculum Preparation

*Didymella bryoniae* fungus (isolate no. 12-003), which is the causal agent of GSB of melon, was collected from the National Institute of Horticultural and Herbal Science (NIHHS), South Korea. Fungus was cultured on petri plates containing 15 mL potato dextrose agar (potato infusion 4 g·L^−1^, dextrose 20 g·L^−1^, agar 15 g·L^−1^). Inoculated plates were then cultured at 24 ± 2 °C under alternating periods of 12 h fluorescent light (40–90 µmol·m^−2^·s^−1^ PPFD) and 12 h darkness for 2–3 weeks until sporulating pycnidia formed. A spore suspension was made by flooding the culture plates with 5–10 mL of sterile distilled water containing Tween-20 (20 drops·L^−1^), which helps to dislodge the spores from mycelia and pycnidia, and by softly scraping the surface of the agar with an L-shaped rubber spreader. The spore suspension was sieved from each plate through a four-layered Mira-cloth (EMD Millipore Corpopration, Burlington, MA, USA) to remove mycelia, pycnidia, and dislodged agar. Finally, the spore suspension was adjusted to a concentration of 5 × 10^5^ spores·mL^−1^ by adding deionized water and with the aid of a hemocytometer.

### 4.4. Inoculation Test for GSB Resistance

A fungal pathogenicity test was performed according to a previously reported method, with slight modifications [9]. Plants at the 4–6 true-leaf stage (3–4 weeks old) were inoculated with the *Didymella bryoniae* spore suspension, near to runoff, using a hand pump spray bottle in a large glasshouse where the maximum and minimum temperature was 25 °C and 20 °C, respectively. Inoculated plants were covered with a plastic tent to maintain high relative humidity (92%). Plants were re-inoculated three days after the first inoculation to ensure no plants had avoided inoculation and to eliminate false positives.

### 4.5. Disease Ratings

**Figure 8:**
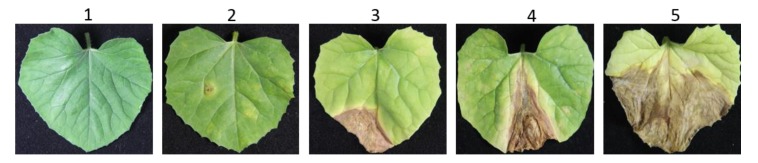
Scale of phenotypic categorization according to the reaction of melon leaves infected with *Didymella bryoniae*. Leaves were rated according to the following scales: 1 = 0% leaf area affected, 2 = 1–10% leaf area affected, 3 = 11–30% leaf area affected, 4 = 31–50% leaf area affected, and 5 = 51–100% leaf area affected.

Inoculated leaves began to show visible symptoms at seven days after inoculation (DAI). However, the final scoring was done at 14 DAI when symptoms were much more prominent. Disease ratings were scored as per the methods stated, with slight modifications [9,10]. Three individual leaves from each inoculated plants were considered for disease rating. Leaves were rated according to the following scales: 1 = 0% of leaf area affected, 2 = 1–10% leaf area affected, 3 = 11–30% leaf area affected, 4 = 31–50% leaf area affected, and 5 = 51–100% (Figure 8). The percentage infected area was measured by the ratio between infected areas by total leaf area, multiplied by 100. The percent disease index (PDI) was measured to reliably identify the phenotype data while using the following equation:

(1) $$\mathrm{PDI}=\;\sum \frac{\mathrm{Sum}\;\mathrm{of}\;\mathrm{numerical}\;\mathrm{disease}\;\mathrm{ratings}}{\mathrm{Number}\;\mathrm{of}\;\mathrm{plants}\;\mathrm{evaluated}\;\times \;\mathrm{maximum}\;\mathrm{disease}\;\mathrm{rating}\;\mathrm{scale}}\;\times \;100$$

Individuals were considered to be resistant if they scored a PDI ≤ 20 (when leaves of the inoculated plants have no symptoms), and susceptible if PDI > 20 (Table S9). 

### 4.6. Exploring GSB Resistance Genes and Primer Design

To date, few studies have explored molecular markers of resistance to GSB in melon. Thus, primers were initially designed using closely related resistance genes from a previously reported SSR marker, CMCT505, which is linked to GSB in melon chromosome 1, and previously reported GSB resistance genes belonging to chromosome 4 [13,14] (Table S10). On the other hand, the stable locus *Gsb5.1*, which is linked to the resistance to GSB, has been repeatedly detected on cucumber chromosome 5 [18]. Therefore, known GSB QTL segments (1.22 Mb) from the cucumber genome were aligned with the melon genome using SyMAP v3.4 to discover GSB resistance genes (Figure 2). A total of 585 putative *R* genes have also been reported in melon throughout all of its chromosomes, of which 40 were present on chromosome 9 [24]. As melon chromosome 9 has reportedly been linked to disease [19], we therefore designed primers from 35 of these 40 *R* genes, which have synteny with cucumber chromosome 5 (Table 2 and Table S11, Figure S8). All of the sequences used in this study were retrieved from the Cucurbit Genomics database (http://cucurbitgenomics.org/search/features).

### 4.7. DNA Extraction

Young leaf samples were collected for DNA isolation from individually tagged, three-week-old melon plants (before pathogen infection) grown in a growth chamber. Genomic DNA was extracted using the DNeasy Plant Mini Kit (QIAGEN, Hidden, Germany), according to the manufacturer’s instructions. The concentration of the extracted DNA was determined using a Nanodrop Spectrophotometer ND-100 (Nanodrop Technologies, Wilmington, DE, USA), and diluted to a working dilution of 50 ng/µL and stored in a refrigerator at −20 °C.

### 4.8. PCR Conditions and Electrophoresis

PCR amplification was done in a 20-µL reaction volume comprising 2 µL of template DNA (50 ng/µL), 1.0 µL (10 pmol) of each forward and reverse primer, 8 µL of 2x Prime Taq Premix (GENET BIO, Nonsan, South Korea), and 8 µL deionized distilled water. The reaction was incubated for initial denaturing at 94 °C for 5 min, followed by 32 cycles at 94 °C for 30 s, 58 °C for 30 s, and 72 °C for 1 min, with a final extension of 72 °C for 7 min. Amplified PCR products were subjected to agarose gel electrophoresis and they were visualized in a gel documentation system.

### 4.9. Cloning and Sequencing

The melon gene MELO3C022157 was PCR-amplified from resistant and susceptible lines using Phusion^®^ High-Fidelity DNA Polymerase (New England Biolabs, EVRY Cedex, France). Amplified DNA fragments were refined using the Promega DNA Purification kit (Promega, Madison, WI, USA), as per the manufacturer’s instructions. Cloning was performed using the TOPO TA cloning kit (Invitrogen, Carlsbad, CA, USA), following the manufacturer’s instructions. The universal primers M13F and M13RpUC were used to sequence the cloned amplicons using an ABI3730XL sequencer (Macrogen Co., Seoul, Korea). Each forward and reverse sequence of resistant and susceptible melon line was repeated five times to remove all ambiguities. Gene sequences between the resistant and susceptible lines were compared using ClustalW omega software (https://www.ebi.ac.uk/Tools/msa/clustalo/) to detect sequence variation.

### 4.10. Statistical Analysis and Software Used

A Chi-square (χ^2^) test for goodness-of-fit was performed to determine deviations of observed data from the expected segregation ratios using Minitab18 statistical software. A value of *p* < 0.05 was considered statistically significant (State College, PA, USA). Primer3plus software was used to design the primers. A physical map of the chromosome was constructed using MapDraw software [42]. The chromosomal synteny distribution of the GSB-linked, stable QTL fragment (*Gsb5.1*) from cucumber chromosome 5 was compared with the genome of melon chromosome 9 while using SyMAP v3.4 and Circos software [43,44].

## 5. Conclusions

The present study revealed that the GSB resistance in melon line ‘PI482399’ is controlled by a single recessive gene. The InDel markers GSB9-kh-1 and GSB9-kh-2 developed in this study, which are linked to GSB resistance and located on melon chromosome 9, showed perfect consistency with a phenotypic assay of the F_2_ population and inbred melon lines. These newly developed InDel markers should accelerate future melon breeding programs.

## Figures and Tables

**Table 1:** Inheritance of gummy stem blight (GSB) resistance in *Cucumis melo*.

Crosses	Generation	Susceptible (PDI > 20)	Resistant (PDI ≤ 20)	Expected Ratio (S:R)	Chi-Square (χ^2^)	*p*
PI482399	P1	0	15			
Cornell ZPPM 339	P2	15	0			
Cornell × PI482399	F_1_	15	0			
Cornell × PI482399	F_2_	126	42	3:1	0.911	0.639

**Table 2:** Melon resistance genes in chromosome 9 and primer specifications.

Sl. No.	Gene ID	Domain	Primer (5’–3’)	Product Length (bp)
1	MELO3C022157	TIR-NBS-LRR	F: ATGGTGCTTGAGAGAATTGG	876
			R: GTTCATGATTGGGGTGAGAA	
2	MELO3C022154	TIR-NBS-LRR	F: CTTTCGAGGCGAAGATACAC	1695
			R: CAGCCTAAGTTGGTGCATTC	
3	MELO3C022152	TIR-NBS-LRR	F: GGCTTCTCCAGCAACAATAA	1703
			R: ATTCCCACATGCCAAGTTT	
4	MELO3C022148	TIR-NBS-LRR	F: GTTGGCAATCTCTCTGGATG	1009
			R: GCATTTGTATCTTCTCATGTGG	
5	MELO3C022146	TIR-NBS-LRR	F: TTTTAGAGGCGAAGATACTCGT	1215
			R: CAAAGCTTGTGGATGTCCTT	
6	MELO3C022145	TIR-NBS-LRR	F: CCACCATCTCTGCTTGAGTT	1915
			R: GTGGAGAAATCATCCACGAC	
7	MELO3C022144	TIR-NBS-LRR	F: TCATCGTCTTCTTTGGATCG	1427
			R: GGACTATAACCAAAACTCTCCA	
8	MELO3C022143	TIR-NBS-LRR	F: GATGCTGCTGATGGACTTCT	1648
			R: CATTCTCCCAAGCTGTGC	
9	MELO3C021485	RLK	F: CCTGAAGATGATATAAGGTGTC	1696
			R: GTGGGAATTCATCATCTGGTTC	
10	MELO3C025516	TIR-NBS-LRR	F: AGTCGAGTCATCGTTACAACA	849
			R: CTCAAGCTCCAAGAGGTTTGTG	
11	MELO3C002877	TIR-NBS-LRR	F: GGCCAAAGAGTTTAGGAATATC	933
			R: ATGATGATTCGACTCCCTGG	
12	MELO3C002928	LRR	F: GCACCAATTTCCATTCATACCC	796
			R: GCCAGGTGGTATTATCCCAGAC	
13	MELO3C005099	RLK	F: TGGTGTTATGGCGGAGGGTTC	775
			R: ATTGAGAACTCTAAGCGAGC	
14	MELO3C005209	LRR	F: GATGCAAAATGCCACCCTGA	949
			R: TGGGCATAATCTCTCCACGGA	
15	MELO3C005236	RLK	F: AGTTGTAGGTAGTGTGCCTG	860
			R: GATTCTGAAGCCTTCCAAGC	
16	MELO3C005267	RLK	F: GCATGTGAAATGTGATTCTCAG	822
			R: GCATTCACTCGAGGATCAC	
17	MELO3C005339	RLK	F: CCTTGTTCTTGGAATGGGGTTG	999
			R: ATCATACTGCTCCACATTCTG	
18	MELO3C005374	RLK	F: CTCTCTGTCAAGTTTCAGCC	1000
			R: AGATTATCACCCACCCATGTAG	
19	MELO3C005413	RLK	F: GAAGGCGGCTTTGGACCCTGA	905
			R: TTCGACGACGGTATTGCGTG	
20	MELO3C005427	LRR	F: ATGATACTGCTTCTTCTACACC	1242
			R: CTGTGAATGTGGAAAGGTCCCA	
21	MELO3C005450	LRR	F: GTCCATAGCAGCAGCAGCTTGC	865
			R: TCCAAACACATCTCCACCAAG	
22	MELO3C005504	LRR	F: ATGGACTCAGAGGCAGGGC	889
			R: GGTCATCAATCTTCTCCAGC	
23	MELO3C005660	RLK	F: AGCCGACAAGAACTTGAATTAGC	721
			R: TGTACGACATCACTTCTGGCA	
24	MELO3C005864	RLK	F: ATGTCAGAGCCGATCAAAGAC	734
			R: GCTAACCCCATTGCAGAACCTCC	
25	MELO3C005732	RLK	F: CCTCACTCTCGCTCTCTTAATTGG	846
			R: GTCAAAAGCATAATGGCACCC	
26	MELO3C025088	RLK	F: ATCGGCCGCCAAAATCTCCAAG	791
			R: GAAAGCAATGTATTCACTCGTG	
27	MELO3C025083	RLK	F: CGGAATCACCACCGGCTCTGTT	1063
			R: CATCTCTTAACCTCTTAACCAC	
28	MELO3C005170	RLK	F: CAACAAGCAAAGACGATTCGAC	1010
			R: GAGAAGGTTCCTATGCACTGCC	
29	MELO3C005428	LRR	F: TCCATAACTGCAGCAGCTTGC	931
			R: CCAAGTGGTTTCCGGAGAGGTC	
30	MELO3C005435	LRR	F: GACCAACCAAATTAAACACCCA	922
			R: ACCTGGCGGCAAAGGACCCGT	
31	MELO3C022083	LRR	F: ATGGGAATGAAGAGAACTGAGG	520
			R: GCTGTGTCATGTTAGCTAAAGAC	
32	MELO3C022040	RLK	F: TCTCAAACACCATCAACGAAGC	1066
			R: GGCAAATCCCACGCTACTACAC	
33	MELO3C005451	LRR	F: ATGATGTTGCTGCTTCTCCCAT	874
			R: GTGGAACTATAATCTTGGTGGAG	
34	MELO3C025517	TIR-NBS-LRR	F: GCATCCTTATCTTCTCCTCCGC	557
			R: GAAGCCGGAGAGAGTGAAAACA	
35	MELO3C005508	LRR	F: ATGGCTTCAACGGAAATCTCAAC	1012
			R: ACCACTCCACAACTTTGACAGT	

**Table 3:** Primer specifications of two InDel markers linked to gummy stem blight in melon.

Gene ID	InDel Marker	Primer (5’–3’)		Product Size
MELO3C022157	Kh-GSB9-1	F	GTTAGGAAACAACAGACCTCCA	266
		R	CAGAACGCACAAAACTCAAAGGAC	
MELO3C022157	Kh-GSB9-2	F	CCTAATAGTCCTTTGAGTTTTGTGCG	409
		R	GGTGTGCTTGGATTGGCTTTCT	

## Supplementary Materials

Supplementary materials can be found at http://www.mdpi.com/1422-0067/19/10/2914/s1.
