# Supplementary material for: Gummy Stem Blight Resistance in Melon: Inheritance Pattern and Development of Molecular Markers
Source: Int J Mol Sci. 2018 Sep 25;19(10):2914. doi: 10.3390/ijms19102914 (PMC6213961; doi:10.3390/ijms19102914)
Supplement: Supplementary file 1 [file ijms-19-02914-s001.zip › Supplementary data/Figure S4.docx]

**A. Genomic DNA sequence**

>MELO3C022157T1 (gDNA) Reference

ATGGAAGCAATTGAGGAATCAAGAACAGCTATTGTGGTTTTATCACAAAACTATTCTACTTCAAGATGGTGCTTGAGAGAATTGGAGAAGATTATGGAATCCATGGACGACGGAACAAATCGAGTTCTTCCTGTGTTTTACCATGTAGATCCTTCTCATGTTCGTCATCAATCTGGACCTTTTGAGAGAAGCTTTGTTGAATATGAAAATAATGGACAAGACTCACAAGAGCAGGTTCATCGGTGGAGGGATGCTTTCGCTAGAGTTGGCCATCTTGCAGGGGTCGTAGTAAACAAAAACAGGTAGGGTCTCACTCTTCTCATTATCATTTTCTTCTTTTTTTTTTATATATAATTATTTCTTCGTATGGTTAGATGTTAGGGAACAACAGACCTCCAAATATCTTCACAATAATATGATATTATCCACTTTAAACATACATCCTTCATGGTTTTCCTTTTGTTTTCACTCAAAAAACTATCTCATATATACCGTTTGAACCAAAAATCCATGGTCATCCCCTTAGTCTTATTTAACCGAAATGTGAAACTTTGATCGCTTTCTCACTTCGCATTAGACAAAATTGGGTGTACTCTAATACCATTTTTGAACCTAATAGTCTTTTGAGTTTTGTGCGTTCTGATACTATACGTTAGTGATAAACATCCTATTTTATCTCAAAAAACCAATTGACTAATGATCAGGAAGCTAGAGGGAGTACTAGGTCATTGTATCTTATCAAACATGCATTTATGAGGTCTCGAACTCCTTTGTTTTTTCCAATGTAGCACACAACCTAGCTCAACACACTTTTATTTTTTAGCATTCAAGTACATAAAATAATATGCGAATAGAAGAAGTTAGAGATTCCTAAATTAATAGCCTAATAAATAGTACTTTAAAAAAATAATATTACACATAATTGTTTCTTTACATATTCCTAGAATCTCTGCTTGTTCTAAAAAGTCTCATGGAATATATAGAAAGGAGCCCGACTAATTGAAGAATTAGATACACAAACTTTTTTAGAATTAGGAAAATAACTCTCTTCATTGATGGTGTGGATTATATAGTGGGGATTCTGTTAATAGTCGTTCATTGTATACTATAGGAAGAAAGAAAGCGAATCCAAGCACACCATAAATTTTTTTTTGACATACTACTCTTTGGCCATGCAGCCCTGAAGTGGACAGTATCAACCGAATCACCAATCAAATATTTGATAAGTTGCGAAGACCTATGTTAATAGGCCCTAATCAATTGAATTACTTGGTTGATATGCGAAGTAAGCTAAGGGATATCAATAACCTACTTGACTTTGAATCAGATGAAGTACGATTTATAGGAATAGTTGGAATGGGTGGTATTGGTAAAACAACTATTGCAAAAGTTTTACACGACAGTATTGCATTTACATTATTTGGTGAAAATTCTTGCTTTGTCACTATGTCTGGGCGTGATATCGTCACGGTCCAATGTCTACTACTCTCTCGACTTCTTGGAACTAGGGAGAATATTAACATTTTAGAAAAGAATGAAGGAGCAAACATGATTAAAGATTGTTTGAGTAGGAGAAAGGTTTTGATTATTTTTGATGGGGTGAATGATAGAGAGGAATTAGGATACATAGCCGGAAGTTTTGATTGGTTTGGTCGAGGAAGTCGAGTCATCATTACCACTAGAAATAAAAATGTTTTTTCTCACCCCAATCATGAACAAGTTCAACTCTACAATGTGAAACCACTTGATTACAACACTTCATTCTCACTTTTTTGGAAGCATGCATTTGATCAACAAAGTGGGGGTCCAAGTGAACAACAATTCATACAACTTAGTCAGAATATAGTGGAAAAGGTCGAAGGAAATCCACAAGCATTGGAACAAATTGGATCATTTTTGCGTGGTAAAGATATTAATGTATGGAAAGAAGAATTGAAGAGCCTTGTTTTAGTTGATAATGAACGTCTCTTCAAAATATTAAAGATAAGTTTTGATCAATTAGGGACAAAAGGCCAACAAGCTTTTCTTGATTTGGCATGTTTCTTCAATGGAAAAAGTACAGGCAAAATTATTGAAATACTTGCGAGTTTAGAATACAATTCCCCCAGCGAAGTACTAAAGTTGTTGTGTGATAGATATCTTATTGAAATTAGAGATGGAGACACAGTATGTATGCCCAATTTGATACAAGAAATGGGTCGAGAAATAGAACGAAAAAAACGTCAAAGAAGCAGGATTTGGCTTAGAAGAGATGCTTTCGACATATTTGATGAACAACATGTAAGTCTATATATGTTACATTATATTATTGTACATTATTTTCCATGTTATTTAATTATATTAACTGATTTACTTTGGCAGGGAGTAAAAGACATAAAAGGTGTTGTGTTGGACAAGAGAGACACAGAACCAAACTTAAAGTTGAAGGCTAAACAATTACAAGATATGAGCCGTTTAAAAATATTAGAGATTGACAATGTGCAGCTGAGTCCAAGAAATCAAAATGATCTCTCAAATCAGCTTCGATTGCTCCACTGGGATGGCTTTCCTTCAGACACTTTGCCACTAAATTTCGAAGCACCATATTTATTTGAACTTCTCTTGCCTAATGCACAAACCACTCATCTTTGGAAAGAACTAAAGGTTAGACATATATTTGCAAATACTTTAACCTGCGCTATATATATATATATGTTGTGATTGTGCTATTTGCAATAATTTTTCAAATTTTCTTTCATAACATATATAAATACATGTTTTGTTATTTGGTAACAGGGATTTAAGAAATTAAAGGTAATCGATGTTAGCAATTCCCAAACTTTGGTGGAGACACCGAATTTGAGTGCTGTTCCAAATCTAGAAAGATTGATTCTATGTAATTGTACAAGATTGAAGAAAATTGATAATTCAATTACAAAATTGAGACTTCTAGTTTTAGTAGACCTCACAGGCTGTGTTCGCCTCGAAACATCCGAGTGCATCGATATCTTGAAGAGTCGCCCAACAGTAGAACTTCGTGGCTTAGTTCTACAGTGTCGCCAATCATTGAAAGGTATTTGTTATATTCGTAAATTTTTTCACTTCTTTTTTAAAATTCAGGATCATTTTGTGTAA

>PI482399 (gDNA) Resistant line

ATGGAAGCAATTGAGGAATCAAGAACAGCTATTGTGGTTTTATCACAAAACTATTCTACTTCAAGATGGTGCTTGAGAGAGTTGGAGAAGATTATGGAATCCATGGACGACGGAACAAATCGAGTTCTTCCTGTGTTTTACCATGTAGATCCTTCTCATGTTCGTCATCAATCTGGACCTTTTGAGAGAAGCTTTGTTGAATATGAAAATAATAGACAATACTCAAGAGAGCAGGTTGGACGGTGGAGGAATGCTTTCAATAGAGTTGGCCAACTTGCAGGGGTCGTAGTAAACAAAAACAGGTAGGGTCTCACTCTTCTCATTTTCATTTTCTTCTTTTTTATATATAATTATTTCTTCATATGGTTAGATGTTAGGAAACAACAGACCTCCAAATATCTTCATAATAGTATGATATTATCCACTTTAAACATACATCCTTCATGATTTTACTTTTGGTTTCACTCAAAAAACCATCTCATATTATACCATTTGAACCAAAAATCCATGGTCATCCCCTTAGTCTTATTTAACCGTACTCTAATACCATTTTTTAACCTAATAGTCCTTTGAGTTTTGTGCGTTCTGATACTATGTTTTTTTTTTTTTCAAATATAGCACACAACCTGGCTCAACACACACTTTTATTTTTTATGGGTTCAAGTACATATAATATAAGTGAATATAGAAAGAAGTTAGAGATTCCTAAATTAATAGCCTAATATATAAATAGTACTTTAAGAAAAATTACACATAATATTGTTTCTTTACATATTCCTTGAATCTCTGCTTGTTCTAAAAAGTCACATGGAATATATAGAAGGAGCCTGACTAATTGAAGAACTAAATACACAAACTTTTTAAAAATAACTCTCTTCATTGATGTGTGGATTATATAGTGGGGATTCTGTTAATAGTCATTCATTGTACACTTAGGAAGAAAGCCAATCCAAGCACACCATAAATTTTTTACATACTACTCTTTGGCCATGCAGCCCTGAAGTGGACAGTATCAACGAAATCTGATCAAATATTTGATAAGTTGCGAAGACCAATGTTAGTAGGCCCTAATCGATCGAATTACTTAGTTGATATGCGAAGTAAGATAAGGGATATCAATAAGCTACTTGACTTTGAATCAGATGAAGTACGATTTATAGGAATAGTTGGAATGGGTGGTATTGGTAAAACAACTATTGCAGAAGTTTTACACGACAGTATTGCATTTACATTGTTTGGCGAAAATTCTTGCTTTGTCACTATGTCTGGACGTGATATCGTCACGGTCCAATGTCTACTACTCTCTCGACTTCTTGGAACTAGGGAGAATATTAACATTTTAGAAAAGAATGAAGGAGCAAACATGATTAAAGATTGTTTGAGTGGGAGAAAGGTTTTGATTATTTTTGATGGGGTGAATGATAGAGAGGAATTAGGATACTTAGCCGGAAGTTTTGATTGGTTTGGTCGAGGAAGTCGAGTCATCATTACCACTAGAAATAAAAATGTTTTTTCTCACCCCAATCATGAACAAGTTCAACTCTACAATGTGAAACCACTTGATTACAACACTTCATTCTCACTTTTTTGGAAGCATGCATTTGATCAACAAAGTGGGGGTCCAAGTGAACAACAATTCATACAACTTAGTCAGAATATAGTGGAAAAGGTCGAAGGAAATCCACAAGCATTGGAACAAATTGGATCATTTTTGCGTGGTAAAGATATTAATGTATGGAAAGAAGAATTGAAGAGCCTTGTTTTAGTTGATAATGAACGTCTCTTCAAAATATTAAAGATAAGTTTTGATCAATTAGGGACAAAAGGCCAACAAGCTTTTCTTGATTTGGCATGTTTCTTCAATGGAAAAAGTACAGGCAAAATTATTGAAATACTTGCGAGTTTAGAATACAATTCCCCCAGCGAAGTACTAAAGTTGTTGTGTGATAGATATCTTATTGAAATTAGAGATGGAGACACAGTATGTATGCCCAATTTGATACAAGAAATGGGTCGAGAAATGGACCGACAAGAACGTCAAAGAAGCAGGATTTGGATTAGAAGAGATGCCTTCGACATATTTGATGAACAACATGTAAGTCGAGAAAAATATATATGTTACATTAGTGTACATTATTTCCATGTTATTTAATTATATTAACTGTTCTATTTTTGGCAGGGAGTAAAAGACATAAAAGGTGTTGTGTTGGACAAGAGAGACACAGAACCAAACTTAAAGTTGAAGGCTAAACAATTACAAAATATGAGCCGTTTAAAAATATTAGAGATTGACAATGTGCAGCTTAGTCCAAGAAATCAAAATGATCTCTCTAATCAGCTTCGATTGCTTCACTGGGATGGCTTTCCTTCAGACACTTTGCCACTAAATTTTGAAGCACCATATTTATTTGAACTTCTCTTGCCTAATGCTCAAACCACTCATCTTTGGAAAGAACTAAAGGTTAGGCATATATTTGCAAATACTTTAACCTGTGCTATATATAGATATATATTTATTTTGTGATTGTTCTATTTGCTATAATTTTTCAAATATTCTTTCATAACATATATAAATACATGTTTTGTTATTTGGTAACAGGGATTTAAGAAATTAAAGGTAATCGATGTTAGCAATTCCCGAACTTTGGTGGAGACACCGAATTTAAGTGCTGTTCCAAATCTAGAAAGATTGATTCTATGTAATTGTACAAGATTGAAGAAAATTGATAATTCAATTACAAAATTGAGACTTCTAGTTTTAGTAGACCTCACAGGCTGTGTTCGCCTCGAAACATCCGAGTGCATCGATATCTTAAAGAGTCGCCCAACAGTAGAACTTCGTGGCTTAGTTCTACAGTGTCACCAATCACCAATCATTAGAAGGTATTTGCTATATTTGTAAATTCTTTCGCTTATCTTTTAAAATTTAGGATCATCTCGTGTAA

>Cornel ZPPM 339 (gDNA) Susceptible line

ATGGAAGCAATTGAGGAATCAAGAACAGCTATTGTGGTTTTATCACAAAACTATTCTACTTCAAGATGGTGCTTGAGAGAATTGGAGAAGATTATGGAATCCATGGACGACGGAACAAATCGAGTTCTTCCTGTGTTTTACCATGTAGATCCTTCTCATGTTCGTCATCAATCTGGACCTTTTGAGAGAAGCTTTGTTGAATATGAAAATAATGGACAAGACTCACAAGAGCAGGTTCATCGGTGGAGGGATGCTTTCGCTAGAGTTGGCCATCTTGCAGGGGTCGTAGTAAACAAAAACAGGTAGGGTCTCACTCTTCTCATTATCATTTTCTTCTTTTTTTTTATATATAATTATTTCTTCGTATGGTTAGATGTTAGGGAACAACAGACCTCCAAATATCTTCACAATAATATGATATTATCCACTTTAAACATACATCCTTCATGGTTTTCCTTTTGTTTTCACTCAAAAAACTATCTCATATATACCGTTTGAACCAAAAATCCATGGTCATCCCCTTAGTCTTATTTAACCGAAATGTGAAACTTTGATCGCTTTCTCACTTCGCATTAGACAAAATTGGGTGTACTCTAATACCATTTTTGAACCTAATAGTCTTTTGAGTTTTGTGCGTTCTGATACTATACGTTAGTGATAAACATCCTATTTTATCTCAAAAAACCAATTGACTAATGATCAGGAAGCTAGAGGGAGTACTAGGTCATTGTATCTTATCAAACATGCATTTATGAGGTCTCGAACTCCTTTGTTTTTTCCAATGTAGCACACAACCTAGCTCAACACACTTTTATTTTTTAGCATTCAAGTACATAAAATAATATGCGAATAGAAGAAGTTAGAGATTCCTAAATTAATAGCCTAATAAATAGTACTTTAAAAAAAAATATTACACATAATTGTTTCTTTACATATTCCTAGAATCTCTGCTTGTTCTAAAAAGTCTCATGGAATATATAGAAAGGAGCCCGACTAATTGAAGAATTAGATACACAAACTTTTTTAGAATTAGGAAAATAACTCTCTTCATTGATGGTGTGGATTATATAGTGGGGATTCTGTTAATAGTCGTTCATTGTATACTATAGGAAGAA**AGAAAGCGAATC**CAAGCACACCATAAATTTTTTTTTGACATACTACTCTTTGGCCATGCAGCCCTGAAGTGGACAGTATCAACCGAATCACCAATCAAATATTTGATAAGTTGCGAAGACCTATGTTAATAGGCCCTAATCAATTGAATTACTTGGTTGATATGCGAAGTAAGCTAAGGGATATCAATAACCTACTTGACTTTGAATCAGATGAAGTACGATTTATAGGAATAGTTGGAATGGGTGGTATTGGTAAAACAACTATTGCAAAAGTTTTACACGACAGTATTGCATTTACATTATTTGGTGAAAATTCTTGCTTTGTCACTATGTCTGGGCGTGATATCGTCACGGTCCAATGTCTACTACTCTCTCGACTTCTTGGAACTAGGGAGAATATTAACATTTTAGAAAAGAATGAAGGAGCAAACATGATTAAAGATTGTTTGAGTAGGAGAAAGGTTTTGATTATTTTTGATGGGGTGAATGATAGAGAGGAATTAGGATACATAGCCGGAAGTTTTGATTGGTTTGGTCGAGGAAGTCGAGTCATCATTACCACTAGAAATAAAAATGTTTTTTCTCACCCCAATCATGAACAAGTTCAACTCTACAATGTGAAACCACTTGATTACAACACTTCATTCTCACTTTTTTGGAAGCATGCATTTGATCAACAAAGTGGGGGTCCAAGTGAACAACAATTCATACAACTTAGTCAGAATATAGTGGAAAAGGTCGAAGGAAATCCACAAGCATTGGAACAAATTGGATCATTTTTGCGTGGTAAAGATATTAATGTATGGAAAGAAGAATTGAAGAGCCTTGTTTTAGTTGATAATGAACGTCTCTTCAAAATATTAAAGATAAGTTTTGATCAATTAGGGACAAAAGGCCAACAAGCTTTTCTTGATTTGGCATGTTTCTTCAATGGAAAAAGTACAGGCAAAATTATTGAAATACTTGCGAGTTTAGAATACAATTCCCCCAGCGAAGTACTAAAGTTGTTGTGTGATAGATATCTTATTGAAATTAGAGATGGAGACACAGTATGTATGCCCAATTTGATACAAGAAATGGGTCGAGAAATAGAACGAAAAAAACGTCAAAGAAGCAGGATTTGGCTTAGAAGAGATGCTTTCGACATATCTGATGAACAACATGTAAGTCTATATATGTTACATTATATTATTGTACATTATTTTCCATGTTATTTAATTATATTAACTGATTTACTTTGGCAGGGAGTAAAAGACATAAAAGGTGTTGTGTTGGACAAGAGAGACACAGAACCAAACTTAAAGTTGAAGGCTAAACAATTACAAGATATGAGCCGTTTAAAAATATTAGAGATTGACAATGTGCAGCTGAGTCCAAGAAATCAAAATGATCTCTCAAATCAGCTTCGATTGCTCCACTGGGATGGCTTTCCTTCAGACACTTTGCCACTAAATTTCGAAGCACCATATTTATTTGAACTTCTCTTGCCTAATGCACAAACCACTCATCTTTGGAAAGAACTAAAGGTTAGACATATATTTGCAAATACTTTAACCTGCGCTATATATATATATATGTTGTGATTGTGCTATTTGCAATAATTTTTCAAATTTTCTTTCATAACATATATAAATACATGTTTTGTTATTTGGTAACAGGGATTTAAGAAATTAAAGGTAATCGATGTTAGCAATTCCCAAACTTTGGTGGAGACACCGAATTTGAGTGCTGTTCCAAATCTAGAAAGATTGATTCTATGTAATTGTACAAGATTGAAGAAAATTGATAATTCAATTACAAAATTGAGACTTCTAGTTTTAGTAGACCTCACAGGCTGTGTTCGCCTCGAAACATCCGAGTGCATCGATATCTTGAAGAGTCGCCCAACAGTAGAACTTCGTGGCTTAGTTCTACAGTGTCGCCAATCATTGAAAGGTATTTGTTATATTCGTAAATTTTTTCACTTCTTTTTTAAAATTCAGGATCATTTTGTGTAA

**gDNA alignment**

PI482399 ATGGAAGCAATTGAGGAATCAAGAACAGCTATTGTGGTTTTATCACAAAACTATTCTACT 60

MELO3C022157T1 ATGGAAGCAATTGAGGAATCAAGAACAGCTATTGTGGTTTTATCACAAAACTATTCTACT 60

Cornel ATGGAAGCAATTGAGGAATCAAGAACAGCTATTGTGGTTTTATCACAAAACTATTCTACT 60

************************************************************

PI482399 TCAAGATGGTGCTTGAGAGAGTTGGAGAAGATTATGGAATCCATGGACGACGGAACAAAT 120

MELO3C022157T1 TCAAGATGGTGCTTGAGAGAATTGGAGAAGATTATGGAATCCATGGACGACGGAACAAAT 120

Cornel TCAAGATGGTGCTTGAGAGAATTGGAGAAGATTATGGAATCCATGGACGACGGAACAAAT 120

********************.***************************************

PI482399 CGAGTTCTTCCTGTGTTTTACCATGTAGATCCTTCTCATGTTCGTCATCAATCTGGACCT 180

MELO3C022157T1 CGAGTTCTTCCTGTGTTTTACCATGTAGATCCTTCTCATGTTCGTCATCAATCTGGACCT 180

Cornel CGAGTTCTTCCTGTGTTTTACCATGTAGATCCTTCTCATGTTCGTCATCAATCTGGACCT 180

************************************************************

PI482399 TTTGAGAGAAGCTTTGTTGAATATGAAAATAATAGACAATACTCAAGAGAGCAGGTTGGA 240

MELO3C022157T1 TTTGAGAGAAGCTTTGTTGAATATGAAAATAATGGACAAGACTCACAAGAGCAGGTTCAT 240

Cornel TTTGAGAGAAGCTTTGTTGAATATGAAAATAATGGACAAGACTCACAAGAGCAGGTTCAT 240

*********************************.***** *****..********** .:

PI482399 CGGTGGAGGAATGCTTTCAATAGAGTTGGCCAACTTGCAGGGGTCGTAGTAAACAAAAAC 300

MELO3C022157T1 CGGTGGAGGGATGCTTTCGCTAGAGTTGGCCATCTTGCAGGGGTCGTAGTAAACAAAAAC 300

Cornel CGGTGGAGGGATGCTTTCGCTAGAGTTGGCCATCTTGCAGGGGTCGTAGTAAACAAAAAC 300

*********.********..************:***************************

PI482399 AGGTAGGGTCTCACTCTTCTCATTTTCATTTTCTTCTTTTTTA----TATATAATTATTT 356

MELO3C022157T1 AGGTAGGGTCTCACTCTTCTCATTATCATTTTCTTCTTTTTTTTTTATATATAATTATTT 360

Cornel AGGTAGGGTCTCACTCTTCTCATTATCATTTTCTTCTTTTTTT-TTATATATAATTATTT 359

************************:*****************: *************

GSB9-kh-1-F

PI482399 CTTCATATGGTTAGATGTTAGGAAACAACAGACCTCCAAATATCTTCATAATAGTATGAT 416

MELO3C022157T1 CTTCGTATGGTTAGATGTTAGGGAACAACAGACCTCCAAATATCTTCACAATAATATGAT 420

Cornel CTTCGTATGGTTAGATGTTAGGGAACAACAGACCTCCAAATATCTTCACAATAATATGAT 419

****.*****************.************************* ****.******

PI482399 ATTATCCACTTTAAACATACATCCTTCATGATTTTACTTTTGGTTTCACTCAAAAAACCA 476

MELO3C022157T1 ATTATCCACTTTAAACATACATCCTTCATGGTTTTCCTTTTGTTTTCACTCAAAAAACTA 480

Cornel ATTATCCACTTTAAACATACATCCTTCATGGTTTTCCTTTTGTTTTCACTCAAAAAACTA 479

******************************.****.****** *************** *

PI482399 TCTCATATTATACCATTTGAACCAAAAATCCATGGTCATCCCCTTAGTCTTATTTAACCG 536

MELO3C022157T1 TCTCATAT-ATACCGTTTGAACCAAAAATCCATGGTCATCCCCTTAGTCTTATTTAACCG 539

Cornel TCTCATAT-ATACCGTTTGAACCAAAAATCCATGGTCATCCCCTTAGTCTTATTTAACCG 538

******** *****.*********************************************

PI482399 T---------------------------------------------------ACTCTAAT 545

MELO3C022157T1 AAATGTGAAACTTTGATCGCTTTCTCACTTCGCATTAGACAAAATTGGGTGTACTCTAAT 599

Cornel AAATGTGAAACTTTGATCGCTTTCTCACTTCGCATTAGACAAAATTGGGTGTACTCTAAT 598

: ********

GSB9-kh-2-F GSB9-kh-1-R

PI482399 ACCATTTTTTAA**CCTAATAGTCCTTTGAGTTTTGTGCG**TTCTGATACTATGTTTTT---- 601

MELO3C022157T1 ACCATTTTTGAACCTAATAGTCTTTTGAGTTTTGTGCGTTCTGATACTATACGTTAGTGA 659

Cornel ACCATTTTTGAACCTAATAGTCTTTTGAGTTTTGTGCGTTCTGATACTATACGTTAGTGA 658

********* ************ ***************************. **:

PI482399 ------------------------------------------------------------ 601

MELO3C022157T1 TAAACATCCTATTTTATCTCAAAAAACCAATTGACTAATGATCAGGAAGCTAGAGGGAGT 719

Cornel TAAACATCCTATTTTATCTCAAAAAACCAATTGACTAATGATCAGGAAGCTAGAGGGAGT 718

PI482399 ----------------------------------------------------TTTTTTTT 609

MELO3C022157T1 ACTAGGTCATTGTATCTTATCAAACATGCATTTATGAGGTCTCGAACTCCTTTGTTTTTT 779

Cornel ACTAGGTCATTGTATCTTATCAAACATGCATTTATGAGGTCTCGAACTCCTTTGTTTTTT 778

* ******

PI482399 CAAATATAGCACACAACCTGGCTCAACACACACTTTTATTTTTTATGGGTTCAAGTACAT 669

MELO3C022157T1 CCAATGTAGCACACAACCTAGCTCAACACACTTTTATT---TTTTAGCATTCAAGTACAT 836

Cornel CCAATGTAGCACACAACCTAGCTCAACACACTTTTATT---TTTTAGCATTCAAGTACAT 835

*.***.*************.***********: **:*: ***::* .***********

PI482399 ATAATATA-AGTGAATATAGAAAGAAGTTAGAGATTCCTAAATTAATAGCCTAATATATA 728

MELO3C022157T1 AAAATAATATGCGA---ATAGAAGAAGTTAGAGATTCCTAAATTAATAGCCTAATAAAT- 892

Cornel AAAATAATATGCGA---ATAGAAGAAGTTAGAGATTCCTAAATTAATAGCCTAATAAAT- 891

*:****:: :* ** ::..***********************************:**

PI482399 AATAGTACTTTAAGAA--AAATTACACATAATATTGTTTCTTTACATATTCCTTGAATCT 786

MELO3C022157T1 ---AGTACTTTAAAAAAATAATATTACACATAATTGTTTCTTTACATATTCCTAGAATCT 949

Cornel ---AGTACTTTAAAAA-AAAATATTACACATAATTGTTTCTTTACATATTCCTAGAATCT 947

**********.** :***:: *** *::*********************:******

PI482399 CTGCTTGTTCTAAAAAGTCACATGGAATATATAGA-AGGAGCCTGACTAATTGAAGAACT 845

MELO3C022157T1 CTGCTTGTTCTAAAAAGTCTCATGGAATATATAGAAAGGAGCCCGACTAATTGAAGAATT 1009

Cornel CTGCTTGTTCTAAAAAGTCTCATGGAATATATAGAAAGGAGCCCGACTAATTGAAGAATT 1007

*******************:*************** ******* ************** *

PI482399 AAATACACAAACTTTTTAA---------AAATAACTCTCTTCATTGAT-GTGTGGATTAT 895

MELO3C022157T1 AGATACACAAACTTTTTTAGAATTAGGAAAATAACTCTCTTCATTGATGGTGTGGATTAT 1069

Cornel AGATACACAAACTTTTTTAGAATTAGGAAAATAACTCTCTTCATTGATGGTGTGGATTAT 1067

*.***************:* ******************** ***********

PI482399 ATAGTGGGGATTCTGTTAATAGTCATTCATTGTACACTTA-----GGAAGAAAGCCAATC 950

MELO3C022157T1 ATAGTGGGGATTCTGTTAATAGTCGTTCATTGTATACTATAGGAAGAAAGAAAGCGAATC 1129

Cornel ATAGTGGGGATTCTGTTAATAGTCGTTCATTGTATACTATAGGAAGAA**AGAAAGCGAATC** 1127

************************.********* ***:: *.******** ****

GSB9-kh-2-R

PI482399 CAAGCACACCATAAATTT----TTTACATACTACTCTTTGGCCATGCAGCCCTGAAGTGG 1006

MELO3C022157T1 CAAGCACACCATAAATTTTTTTTTGACATACTACTCTTTGGCCATGCAGCCCTGAAGTGG 1189

Cornel **CAAGCACACC**ATAAATTTTTTTTTGACATACTACTCTTTGGCCATGCAGCCCTGAAGTGG 1187

****************** ** ***********************************

PI482399 ACAGTATCAACGAAATC--TGATCAAATATTTGATAAGTTGCGAAGACCAATGTTAGTAG 1064

MELO3C022157T1 ACAGTATCAACCGAATCACCAATCAAATATTTGATAAGTTGCGAAGACCTATGTTAATAG 1249

Cornel ACAGTATCAACCGAATCACCAATCAAATATTTGATAAGTTGCGAAGACCTATGTTAATAG 1247

*********** .**** .****************************:******.***

**2-bp deletion (2^nd^ exon)**

PI482399 GCCCTAATCGATCGAATTACTTAGTTGATATGCGAAGTAAGATAAGGGATATCAATAAGC 1124

MELO3C022157T1 GCCCTAATCAATTGAATTACTTGGTTGATATGCGAAGTAAGCTAAGGGATATCAATAACC 1309

Cornel GCCCTAATCAATTGAATTACTTGGTTGATATGCGAAGTAAGCTAAGGGATATCAATAACC 1307

*********.** *********.******************.**************** *

PI482399 TACTTGACTTTGAATCAGATGAAGTACGATTTATAGGAATAGTTGGAATGGGTGGTATTG 1184

MELO3C022157T1 TACTTGACTTTGAATCAGATGAAGTACGATTTATAGGAATAGTTGGAATGGGTGGTATTG 1369

Cornel TACTTGACTTTGAATCAGATGAAGTACGATTTATAGGAATAGTTGGAATGGGTGGTATTG 1367

************************************************************

PI482399 GTAAAACAACTATTGCAGAAGTTTTACACGACAGTATTGCATTTACATTGTTTGGCGAAA 1244

MELO3C022157T1 GTAAAACAACTATTGCAAAAGTTTTACACGACAGTATTGCATTTACATTATTTGGTGAAA 1429

Cornel GTAAAACAACTATTGCAAAAGTTTTACACGACAGTATTGCATTTACATTATTTGGTGAAA 1427

*****************.*******************************.***** ****

PI482399 ATTCTTGCTTTGTCACTATGTCTGGACGTGATATCGTCACGGTCCAATGTCTACTACTCT 1304

MELO3C022157T1 ATTCTTGCTTTGTCACTATGTCTGGGCGTGATATCGTCACGGTCCAATGTCTACTACTCT 1489

Cornel ATTCTTGCTTTGTCACTATGTCTGGGCGTGATATCGTCACGGTCCAATGTCTACTACTCT 1487

*************************.**********************************

PI482399 CTCGACTTCTTGGAACTAGGGAGAATATTAACATTTTAGAAAAGAATGAAGGAGCAAACA 1364

MELO3C022157T1 CTCGACTTCTTGGAACTAGGGAGAATATTAACATTTTAGAAAAGAATGAAGGAGCAAACA 1549

Cornel CTCGACTTCTTGGAACTAGGGAGAATATTAACATTTTAGAAAAGAATGAAGGAGCAAACA 1547

************************************************************

PI482399 TGATTAAAGATTGTTTGAGTGGGAGAAAGGTTTTGATTATTTTTGATGGGGTGAATGATA 1424

MELO3C022157T1 TGATTAAAGATTGTTTGAGTAGGAGAAAGGTTTTGATTATTTTTGATGGGGTGAATGATA 1609

Cornel TGATTAAAGATTGTTTGAGTAGGAGAAAGGTTTTGATTATTTTTGATGGGGTGAATGATA 1607

********************.***************************************

PI482399 GAGAGGAATTAGGATACTTAGCCGGAAGTTTTGATTGGTTTGGTCGAGGAAGTCGAGTCA 1484

MELO3C022157T1 GAGAGGAATTAGGATACATAGCCGGAAGTTTTGATTGGTTTGGTCGAGGAAGTCGAGTCA 1669

Cornel GAGAGGAATTAGGATACATAGCCGGAAGTTTTGATTGGTTTGGTCGAGGAAGTCGAGTCA 1667

*****************:******************************************

PI482399 TCATTACCACTAGAAATAAAAATGTTTTTTCTCACCCCAATCATGAACAAGTTCAACTCT 1544

MELO3C022157T1 TCATTACCACTAGAAATAAAAATGTTTTTTCTCACCCCAATCATGAACAAGTTCAACTCT 1729

Cornel TCATTACCACTAGAAATAAAAATGTTTTTTCTCACCCCAATCATGAACAAGTTCAACTCT 1727

************************************************************

PI482399 ACAATGTGAAACCACTTGATTACAACACTTCATTCTCACTTTTTTGGAAGCATGCATTTG 1604

MELO3C022157T1 ACAATGTGAAACCACTTGATTACAACACTTCATTCTCACTTTTTTGGAAGCATGCATTTG 1789

Cornel ACAATGTGAAACCACTTGATTACAACACTTCATTCTCACTTTTTTGGAAGCATGCATTTG 1787

************************************************************

PI482399 ATCAACAAAGTGGGGGTCCAAGTGAACAACAATTCATACAACTTAGTCAGAATATAGTGG 1664

MELO3C022157T1 ATCAACAAAGTGGGGGTCCAAGTGAACAACAATTCATACAACTTAGTCAGAATATAGTGG 1849

Cornel ATCAACAAAGTGGGGGTCCAAGTGAACAACAATTCATACAACTTAGTCAGAATATAGTGG 1847

************************************************************

PI482399 AAAAGGTCGAAGGAAATCCACAAGCATTGGAACAAATTGGATCATTTTTGCGTGGTAAAG 1724

MELO3C022157T1 AAAAGGTCGAAGGAAATCCACAAGCATTGGAACAAATTGGATCATTTTTGCGTGGTAAAG 1909

Cornel AAAAGGTCGAAGGAAATCCACAAGCATTGGAACAAATTGGATCATTTTTGCGTGGTAAAG 1907

************************************************************

PI482399 ATATTAATGTATGGAAAGAAGAATTGAAGAGCCTTGTTTTAGTTGATAATGAACGTCTCT 1784

MELO3C022157T1 ATATTAATGTATGGAAAGAAGAATTGAAGAGCCTTGTTTTAGTTGATAATGAACGTCTCT 1969

Cornel ATATTAATGTATGGAAAGAAGAATTGAAGAGCCTTGTTTTAGTTGATAATGAACGTCTCT 1967

************************************************************

PI482399 TCAAAATATTAAAGATAAGTTTTGATCAATTAGGGACAAAAGGCCAACAAGCTTTTCTTG 1844

MELO3C022157T1 TCAAAATATTAAAGATAAGTTTTGATCAATTAGGGACAAAAGGCCAACAAGCTTTTCTTG 2029

Cornel TCAAAATATTAAAGATAAGTTTTGATCAATTAGGGACAAAAGGCCAACAAGCTTTTCTTG 2027

************************************************************

PI482399 ATTTGGCATGTTTCTTCAATGGAAAAAGTACAGGCAAAATTATTGAAATACTTGCGAGTT 1904

MELO3C022157T1 ATTTGGCATGTTTCTTCAATGGAAAAAGTACAGGCAAAATTATTGAAATACTTGCGAGTT 2089

Cornel ATTTGGCATGTTTCTTCAATGGAAAAAGTACAGGCAAAATTATTGAAATACTTGCGAGTT 2087

************************************************************

PI482399 TAGAATACAATTCCCCCAGCGAAGTACTAAAGTTGTTGTGTGATAGATATCTTATTGAAA 1964

MELO3C022157T1 TAGAATACAATTCCCCCAGCGAAGTACTAAAGTTGTTGTGTGATAGATATCTTATTGAAA 2149

Cornel TAGAATACAATTCCCCCAGCGAAGTACTAAAGTTGTTGTGTGATAGATATCTTATTGAAA 2147

************************************************************

PI482399 TTAGAGATGGAGACACAGTATGTATGCCCAATTTGATACAAGAAATGGGTCGAGAAATGG 2024

MELO3C022157T1 TTAGAGATGGAGACACAGTATGTATGCCCAATTTGATACAAGAAATGGGTCGAGAAATAG 2209

Cornel TTAGAGATGGAGACACAGTATGTATGCCCAATTTGATACAAGAAATGGGTCGAGAAATAG 2207

**********************************************************.*

PI482399 ACCGACAAGAACGTCAAAGAAGCAGGATTTGGATTAGAAGAGATGCCTTCGACATATTTG 2084

MELO3C022157T1 AACGAAAAAAACGTCAAAGAAGCAGGATTTGGCTTAGAAGAGATGCTTTCGACATATTTG 2269

Cornel AACGAAAAAAACGTCAAAGAAGCAGGATTTGGCTTAGAAGAGATGCTTTCGACATATCTG 2267

*.***.**.***********************.************* ********** **

PI482399 ATGAACAACATGTAAGTCGAGAAAAATATATATGTTACATTAGTGTACATT-ATTTCCAT 2143

MELO3C022157T1 ATGAACAACATGTAAGTCTATATATGTTAC---ATTATATTATTGTACATTATTTTCCAT 2326

Cornel ATGAACAACATGTAAGTCTATATATGTTAC---ATTATATTATTGTACATTATTTTCCAT 2324

****************** * *:*:.*::. .*** **** ******** :*******

PI482399 GTTATTTAATTATATTAACTGTTCTATTTTTGGCAGGGAGTAAAAGACATAAAAGGTGTT 2203

MELO3C022157T1 GTTATTTAATTATATTAACTGATTT-ACTTTGGCAGGGAGTAAAAGACATAAAAGGTGTT 2385

Cornel GTTATTTAATTATATTAACTGATTT-ACTTTGGCAGGGAGTAAAAGACATAAAAGGTGTT 2383

*********************:* * : ********************************

PI482399 GTGTTGGACAAGAGAGACACAGAACCAAACTTAAAGTTGAAGGCTAAACAATTACAAAAT 2263

MELO3C022157T1 GTGTTGGACAAGAGAGACACAGAACCAAACTTAAAGTTGAAGGCTAAACAATTACAAGAT 2445

Cornel GTGTTGGACAAGAGAGACACAGAACCAAACTTAAAGTTGAAGGCTAAACAATTACAAGAT 2443

*********************************************************.**

PI482399 ATGAGCCGTTTAAAAATATTAGAGATTGACAATGTGCAGCTTAGTCCAAGAAATCAAAAT 2323

MELO3C022157T1 ATGAGCCGTTTAAAAATATTAGAGATTGACAATGTGCAGCTGAGTCCAAGAAATCAAAAT 2505

Cornel ATGAGCCGTTTAAAAATATTAGAGATTGACAATGTGCAGCTGAGTCCAAGAAATCAAAAT 2503

***************************************** ******************

PI482399 GATCTCTCTAATCAGCTTCGATTGCTTCACTGGGATGGCTTTCCTTCAGACACTTTGCCA 2383

MELO3C022157T1 GATCTCTCAAATCAGCTTCGATTGCTCCACTGGGATGGCTTTCCTTCAGACACTTTGCCA 2565

Cornel GATCTCTCAAATCAGCTTCGATTGCTCCACTGGGATGGCTTTCCTTCAGACACTTTGCCA 2563

********:***************** *********************************

PI482399 CTAAATTTTGAAGCACCATATTTATTTGAACTTCTCTTGCCTAATGCTCAAACCACTCAT 2443

MELO3C022157T1 CTAAATTTCGAAGCACCATATTTATTTGAACTTCTCTTGCCTAATGCACAAACCACTCAT 2625

Cornel CTAAATTTCGAAGCACCATATTTATTTGAACTTCTCTTGCCTAATGCACAAACCACTCAT 2623

******** **************************************:************

PI482399 CTTTGGAAAGAACTAAAGGTTAGGCATATATTTGCAAATACTTTAACCTGTGCTATATAT 2503

MELO3C022157T1 CTTTGGAAAGAACTAAAGGTTAGACATATATTTGCAAATACTTTAACCTGCGCTATATAT 2685

Cornel CTTTGGAAAGAACTAAAGGTTAGACATATATTTGCAAATACTTTAACCTGCGCTATATAT 2683

***********************.************************** *********

PI482399 AGATATATATTTATTTTGTGATTGTTCTATTTGCTATAATTTTTCAAATATTCTTTCATA 2563

MELO3C022157T1 ATA------TATATGTTGTGATTGTGCTATTTGCAATAATTTTTCAAATTTTCTTTCATA 2739

Cornel ATA------TATATGTTGTGATTGTGCTATTTGCAATAATTTTTCAAATTTTCTTTCATA 2737

* * *:*** ********** ********:**************:**********

PI482399 ACATATATAAATACATGTTTTGTTATTTGGTAACAGGGATTTAAGAAATTAAAGGTAATC 2623

MELO3C022157T1 ACATATATAAATACATGTTTTGTTATTTGGTAACAGGGATTTAAGAAATTAAAGGTAATC 2799

Cornel ACATATATAAATACATGTTTTGTTATTTGGTAACAGGGATTTAAGAAATTAAAGGTAATC 2797

************************************************************

PI482399 GATGTTAGCAATTCCCGAACTTTGGTGGAGACACCGAATTTAAGTGCTGTTCCAAATCTA 2683

MELO3C022157T1 GATGTTAGCAATTCCCAAACTTTGGTGGAGACACCGAATTTGAGTGCTGTTCCAAATCTA 2859

Cornel GATGTTAGCAATTCCCAAACTTTGGTGGAGACACCGAATTTGAGTGCTGTTCCAAATCTA 2857

****************.************************.******************

PI482399 GAAAGATTGATTCTATGTAATTGTACAAGATTGAAGAAAATTGATAATTCAATTACAAAA 2743

MELO3C022157T1 GAAAGATTGATTCTATGTAATTGTACAAGATTGAAGAAAATTGATAATTCAATTACAAAA 2919

Cornel GAAAGATTGATTCTATGTAATTGTACAAGATTGAAGAAAATTGATAATTCAATTACAAAA 2917

************************************************************

PI482399 TTGAGACTTCTAGTTTTAGTAGACCTCACAGGCTGTGTTCGCCTCGAAACATCCGAGTGC 2803

MELO3C022157T1 TTGAGACTTCTAGTTTTAGTAGACCTCACAGGCTGTGTTCGCCTCGAAACATCCGAGTGC 2979

Cornel TTGAGACTTCTAGTTTTAGTAGACCTCACAGGCTGTGTTCGCCTCGAAACATCCGAGTGC 2977

************************************************************

PI482399 ATCGATATCTTAAAGAGTCGCCCAACAGTAGAACTTCGTGGCTTAGTTCTACAGTGTCAC 2863

MELO3C022157T1 ATCGATATCTTGAAGAGTCGCCCAACAGTAGAACTTCGTGGCTTAGTTCTACAGTGTCGC 3039

Cornel ATCGATATCTTGAAGAGTCGCCCAACAGTAGAACTTCGTGGCTTAGTTCTACAGTGTCGC 3037

***********.**********************************************.*

PI482399 CAATCACCAATCATTAGAAGGTATTTGCTATATTTGTAAATTCTTTCGCTTATCTTTTAA 2923

MELO3C022157T1 CAATC-------ATTGAAAGGTATTTGTTATATTCGTAAATTTTTTCACTTCTTTTTTAA 3092

Cornel CAATC-------ATTGAAAGGTATTTGTTATATTCGTAAATTTTTTCACTTCTTTTTTAA 3090

***** ***..********** ****** ******* ****.***.* ******

**7-bp insertion (4^th^ exon)**

PI482399 AATTTAGGATCATCTCGTGTAA 2945

MELO3C022157T1 AATTCAGGATCATTTTGTGTAA 3114

Cornel AATTCAGGATCATTTTGTGTAA 3112

**** ******** * ******

**B. ORF analysis**

**PI482399 (resistant line)**


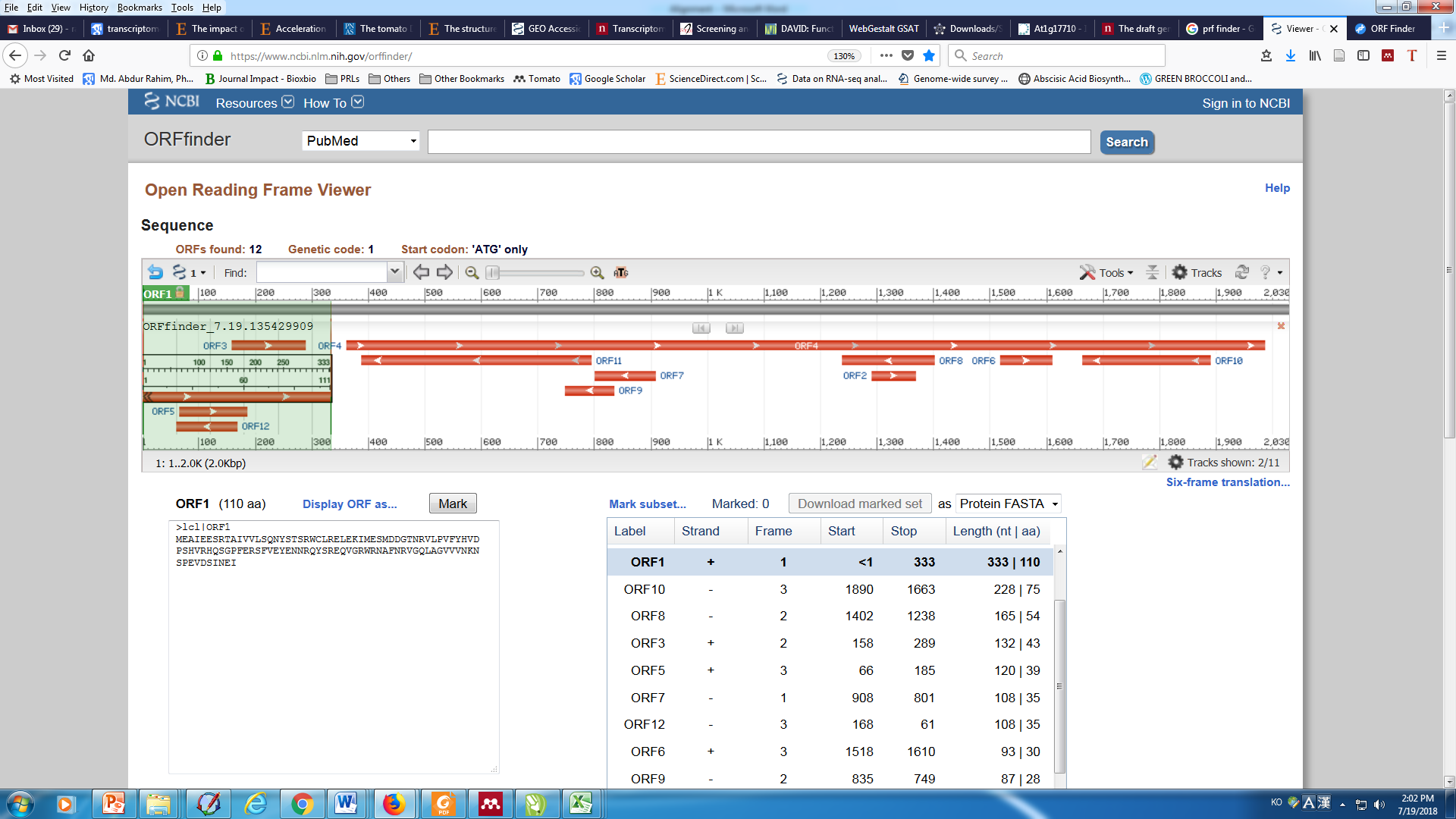


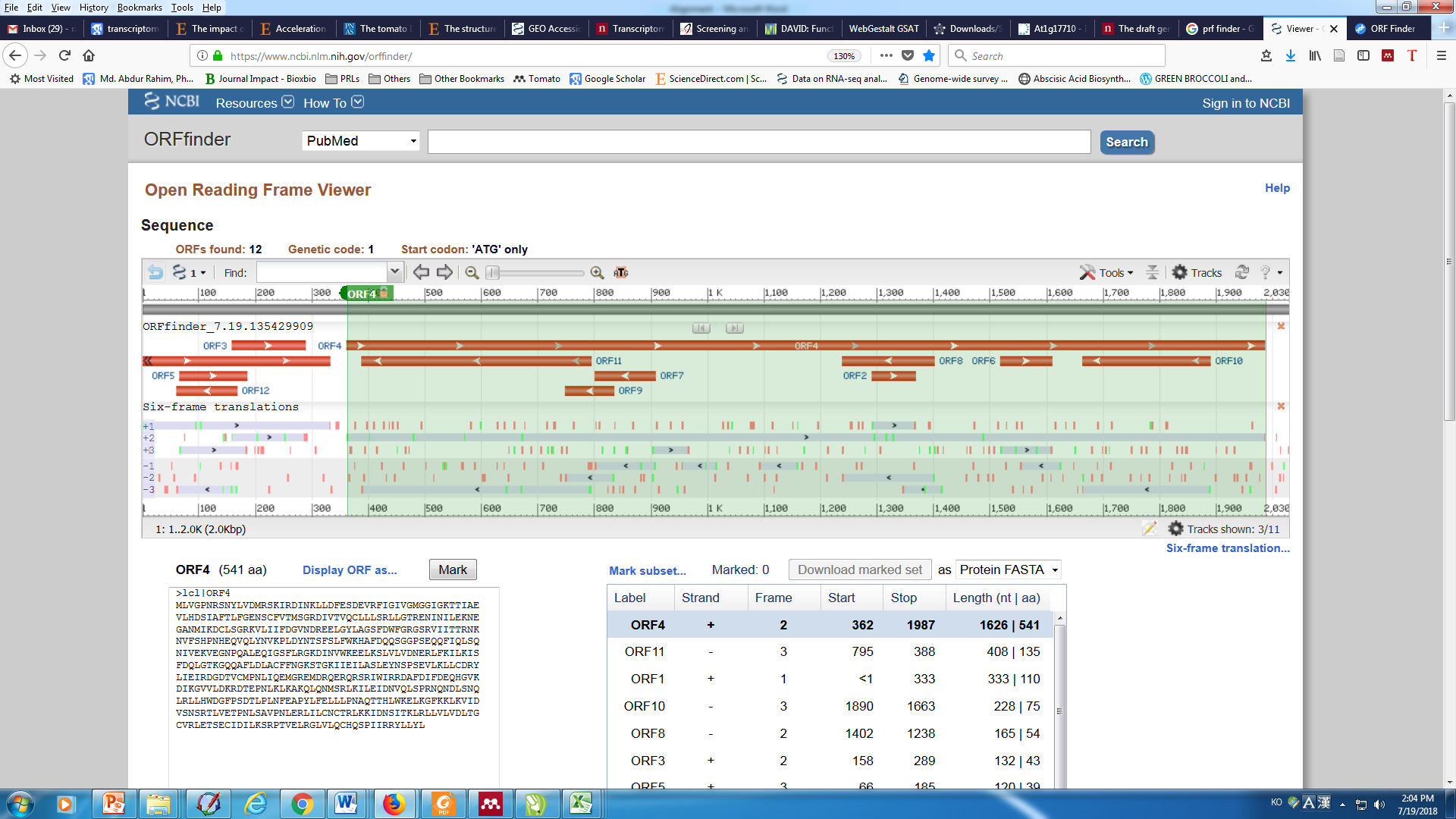


**Open Reading Frame 1 (grey highlighted)**

1 M E A I E E S R T A I V V L S Q N Y S T

1 ATGGAAGCAATTGAGGAATCAAGAACAGCTATTGTGGTTTTATCACAAAACTATTCTACT

1 10 20 30 40 50

1 TACCTTCGTTAACTCCTTAGTTCTTGTCGATAACACCAAAATAGTGTTTTGATAAGATGA

21 S R W C L R E L E K I M E S M D D G T N

61 TCAAGATGGTGCTTGAGAGAGTTGGAGAAGATTATGGAATCCATGGACGACGGAACAAAT

61 70 80 90 100 110

61 AGTTCTACCACGAACTCTCTCAACCTCTTCTAATACCTTAGGTACCTGCTGCCTTGTTTA

41 R V L P V F Y H V D P S H V R H Q S G P

121 CGAGTTCTTCCTGTGTTTTACCATGTAGATCCTTCTCATGTTCGTCATCAATCTGGACCT

121 130 140 150 160 170

121 GCTCAAGAAGGACACAAAATGGTACATCTAGGAAGAGTACAAGCAGTAGTTAGACCTGGA

61 F E R S F V E Y E N N R Q Y S R E Q V G

181 TTTGAGAGAAGCTTTGTTGAATATGAAAATAATAGACAATACTCAAGAGAGCAGGTTGGA

181 190 200 210 220 230

181 AAACTCTCTTCGAAACAACTTATACTTTTATTATCTGTTATGAGTTCTCTCGTCCAACCT

81 R W R N A F N R V G Q L A G V V V N K N

241 CGGTGGAGGAATGCTTTCAATAGAGTTGGCCAACTTGCAGGGGTCGTAGTAAACAAAAAC

241 250 260 270 280 290

241 GCCACCTCCTTACGAAAGTTATCTCAACCGGTTGAACGTCCCCAGCATCATTTGTTTTTG

101 S P E V D S I N E I * S N I * * V A K T

301 AGCCCTGAAGTGGACAGTATCAACGAAATCTGATCAAATATTTGATAAGTTGCGAAGACC

301 310 320 330 340 350

301 TCGGGACTTCACCTGTCATAGTTGCTTTAGACTAGTTTATAAACTATTCAACGCTTCTGG

121 N V S R P * S I E L L S * Y A K * D K G

361 AATGTTAGTAGGCCCTAATCGATCGAATTACTTAGTTGATATGCGAAGTAAGATAAGGGA

361 370 380 390 400 410

361 TTACAATCATCCGGGATTAGCTAGCTTAATGAATCAACTATACGCTTCATTCTATTCCCT

141 Y Q * A T * L * I R * S T I Y R N S W N

421 TATCAATAAGCTACTTGACTTTGAATCAGATGAAGTACGATTTATAGGAATAGTTGGAAT

421 430 440 450 460 470

421 ATAGTTATTCGATGAACTGAAACTTAGTCTACTTCATGCTAAATATCCTTATCAACCTTA

161 G W Y W * N N Y C R S F T R Q Y C I Y I

481 GGGTGGTATTGGTAAAACAACTATTGCAGAAGTTTTACACGACAGTATTGCATTTACATT

481 490 500 510 520 530

481 CCCACCATAACCATTTTGTTGATAACGTCTTCAAAATGTGCTGTCATAACGTAAATGTAA

181 V W R K F L L C H Y V W T * Y R H G P M

541 GTTTGGCGAAAATTCTTGCTTTGTCACTATGTCTGGACGTGATATCGTCACGGTCCAATG

541 550 560 570 580 590

541 CAAACCGCTTTTAAGAACGAAACAGTGATACAGACCTGCACTATAGCAGTGCCAGGTTAC

201 S T T L S T S W N * G E Y * H F R K E *

601 TCTACTACTCTCTCGACTTCTTGGAACTAGGGAGAATATTAACATTTTAGAAAAGAATGA

601 610 620 630 640 650

601 AGATGATGAGAGAGCTGAAGAACCTTGATCCCTCTTATAATTGTAAAATCTTTTCTTACT

221 R S K H D * R L F E W E K G F D Y F * W

661 AGGAGCAAACATGATTAAAGATTGTTTGAGTGGGAGAAAGGTTTTGATTATTTTTGATGG

661 670 680 690 700 710

661 TCCTCGTTTGTACTAATTTCTAACAAACTCACCCTCTTTCCAAAACTAATAAAAACTACC

241 G E * * R G I R I L S R K F * L V W S R

721 GGTGAATGATAGAGAGGAATTAGGATACTTAGCCGGAAGTTTTGATTGGTTTGGTCGAGG

721 730 740 750 760 770

721 CCACTTACTATCTCTCCTTAATCCTATGAATCGGCCTTCAAAACTAACCAAACCAGCTCC

261 K S S H H Y H * K * K C F F S P Q S * T

781 AAGTCGAGTCATCATTACCACTAGAAATAAAAATGTTTTTTCTCACCCCAATCATGAACA

781 790 800 810 820 830

781 TTCAGCTCAGTAGTAATGGTGATCTTTATTTTTACAAAAAAGAGTGGGGTTAGTACTTGT

281 S S T L Q C E T T * L Q H F I L T F L E

841 AGTTCAACTCTACAATGTGAAACCACTTGATTACAACACTTCATTCTCACTTTTTTGGAA

841 850 860 870 880 890

841 TCAAGTTGAGATGTTACACTTTGGTGAACTAATGTTGTGAAGTAAGAGTGAAAAAACCTT

301 A C I * S T K W G S K * T T I H T T * S

901 GCATGCATTTGATCAACAAAGTGGGGGTCCAAGTGAACAACAATTCATACAACTTAGTCA

901 910 920 930 940 950

901 CGTACGTAAACTAGTTGTTTCACCCCCAGGTTCACTTGTTGTTAAGTATGTTGAATCAGT

321 E Y S G K G R R K S T S I G T N W I I F

961 GAATATAGTGGAAAAGGTCGAAGGAAATCCACAAGCATTGGAACAAATTGGATCATTTTT

961 970 980 990 1000 1010

961 CTTATATCACCTTTTCCAGCTTCCTTTAGGTGTTCGTAACCTTGTTTAACCTAGTAAAAA

341 A W * R Y * C M E R R I E E P C F S * *

1021 GCGTGGTAAAGATATTAATGTATGGAAAGAAGAATTGAAGAGCCTTGTTTTAGTTGATAA

1021 1030 1040 1050 1060 1070

1021 CGCACCATTTCTATAATTACATACCTTTCTTCTTAACTTCTCGGAACAAAATCAACTATT

361 * T S L Q N I K D K F * S I R D K R P T

1081 TGAACGTCTCTTCAAAATATTAAAGATAAGTTTTGATCAATTAGGGACAAAAGGCCAACA

1081 1090 1100 1110 1120 1130

1081 ACTTGCAGAGAAGTTTTATAATTTCTATTCAAAACTAGTTAATCCCTGTTTTCCGGTTGT

381 S F S * F G M F L Q W K K Y R Q N Y * N

1141 AGCTTTTCTTGATTTGGCATGTTTCTTCAATGGAAAAAGTACAGGCAAAATTATTGAAAT

1141 1150 1160 1170 1180 1190

1141 TCGAAAAGAACTAAACCGTACAAAGAAGTTACCTTTTTCATGTCCGTTTTAATAACTTTA

401 T C E F R I Q F P Q R S T K V V V * * I

1201 ACTTGCGAGTTTAGAATACAATTCCCCCAGCGAAGTACTAAAGTTGTTGTGTGATAGATA

1201 1210 1220 1230 1240 1250

1201 TGAACGCTCAAATCTTATGTTAAGGGGGTCGCTTCATGATTTCAACAACACACTATCTAT

421 S Y * N * R W R H S M Y A Q F D T R N G

1261 TCTTATTGAAATTAGAGATGGAGACACAGTATGTATGCCCAATTTGATACAAGAAATGGG

1261 1270 1280 1290 1300 1310

1261 AGAATAACTTTAATCTCTACCTCTGTGTCATACATACGGGTTAAACTATGTTCTTTACCC

441 S R N G P T R T S K K Q D L D * K R C L

1321 TCGAGAAATGGACCGACAAGAACGTCAAAGAAGCAGGATTTGGATTAGAAGAGATGCCTT

1321 1330 1340 1350 1360 1370

1321 AGCTCTTTACCTGGCTGTTCTTGCAGTTTCTTCGTCCTAAACCTAATCTTCTCTACGGAA

461 R H I * * T T W S K R H K R C C V G Q E

1381 CGACATATTTGATGAACAACATGGAGTAAAAGACATAAAAGGTGTTGTGTTGGACAAGAG

1381 1390 1400 1410 1420 1430

1381 GCTGTATAAACTACTTGTTGTACCTCATTTTCTGTATTTTCCACAACACAACCTGTTCTC

481 R H R T K L K V E G * T I T K Y E P F K

1441 AGACACAGAACCAAACTTAAAGTTGAAGGCTAAACAATTACAAAATATGAGCCGTTTAAA

1441 1450 1460 1470 1480 1490

1441 TCTGTGTCTTGGTTTGAATTTCAACTTCCGATTTGTTAATGTTTTATACTCGGCAAATTT

501 N I R D * Q C A A * S K K S K * S L * S

1501 AATATTAGAGATTGACAATGTGCAGCTTAGTCCAAGAAATCAAAATGATCTCTCTAATCA

1501 1510 1520 1530 1540 1550

1501 TTATAATCTCTAACTGTTACACGTCGAATCAGGTTCTTTAGTTTTACTAGAGAGATTAGT

521 A S I A S L G W L S F R H F A T K F * S

1561 GCTTCGATTGCTTCACTGGGATGGCTTTCCTTCAGACACTTTGCCACTAAATTTTGAAGC

1561 1570 1580 1590 1600 1610

1561 CGAAGCTAACGAAGTGACCCTACCGAAAGGAAGTCTGTGAAACGGTGATTTAAAACTTCG

541 T I F I * T S L A * C S N H S S L E R T

1621 ACCATATTTATTTGAACTTCTCTTGCCTAATGCTCAAACCACTCATCTTTGGAAAGAACT

1621 1630 1640 1650 1660 1670

1621 TGGTATAAATAAACTTGAAGAGAACGGATTACGAGTTTGGTGAGTAGAAACCTTTCTTGA

561 K G I * E I K G N R C * Q F P N F G G D

1681 AAAGGGATTTAAGAAATTAAAGGTAATCGATGTTAGCAATTCCCGAACTTTGGTGGAGAC

1681 1690 1700 1710 1720 1730

1681 TTTCCCTAAATTCTTTAATTTCCATTAGCTACAATCGTTAAGGGCTTGAAACCACCTCTG

581 T E F K C C S K S R K I D S M * L Y K I

1741 ACCGAATTTAAGTGCTGTTCCAAATCTAGAAAGATTGATTCTATGTAATTGTACAAGATT

1741 1750 1760 1770 1780 1790

1741 TGGCTTAAATTCACGACAAGGTTTAGATCTTTCTAACTAAGATACATTAACATGTTCTAA

601 E E N * * F N Y K I E T S S F S R P H R

1801 GAAGAAAATTGATAATTCAATTACAAAATTGAGACTTCTAGTTTTAGTAGACCTCACAGG

1801 1810 1820 1830 1840 1850

1801 CTTCTTTTAACTATTAAGTTAATGTTTTAACTCTGAAGATCAAAATCATCTGGAGTGTCC

621 L C S P R N I R V H R Y L K E S P N S R

1861 CTGTGTTCGCCTCGAAACATCCGAGTGCATCGATATCTTAAAGAGTCGCCCAACAGTAGA

1861 1870 1880 1890 1900 1910

1861 GACACAAGCGGAGCTTTGTAGGCTCACGTAGCTATAGAATTTCTCAGCGGGTTGTCATCT

641 T S W L S S T V S P I T N H * K V F A I

1921 ACTTCGTGGCTTAGTTCTACAGTGTCACCAATCACCAATCATTAGAAGGTATTTGCTATA

1921 1930 1940 1950 1960 1970

1921 TGAAGCACCGAATCAAGATGTCACAGTGGTTAGTGGTTAGTAATCTTCCATAAACGATAT

661 F V N S F A Y L L K F R I I S C

1981 TTTGTAAATTCTTTCGCTTATCTTTTAAAATTTAGGATCATCTCGTGTAA

1981 1990 2000 2010 2020

1981 AAACATTTAAGAAAGCGAATAGAAAATTTTAAATCCTAGTAGAGCACATT

**Open Reading Frame 4 (grey highlighted)**

1 W K Q L R N Q E Q L L W F Y H K T I L L

1 ATGGAAGCAATTGAGGAATCAAGAACAGCTATTGTGGTTTTATCACAAAACTATTCTACT

1 10 20 30 40 50

1 TACCTTCGTTAACTCCTTAGTTCTTGTCGATAACACCAAAATAGTGTTTTGATAAGATGA

21 Q D G A * E S W R R L W N P W T T E Q I

61 TCAAGATGGTGCTTGAGAGAGTTGGAGAAGATTATGGAATCCATGGACGACGGAACAAAT

61 70 80 90 100 110

61 AGTTCTACCACGAACTCTCTCAACCTCTTCTAATACCTTAGGTACCTGCTGCCTTGTTTA

41 E F F L C F T M * I L L M F V I N L D L

121 CGAGTTCTTCCTGTGTTTTACCATGTAGATCCTTCTCATGTTCGTCATCAATCTGGACCT

121 130 140 150 160 170

121 GCTCAAGAAGGACACAAAATGGTACATCTAGGAAGAGTACAAGCAGTAGTTAGACCTGGA

61 L R E A L L N M K I I D N T Q E S R L D

181 TTTGAGAGAAGCTTTGTTGAATATGAAAATAATAGACAATACTCAAGAGAGCAGGTTGGA

181 190 200 210 220 230

181 AAACTCTCTTCGAAACAACTTATACTTTTATTATCTGTTATGAGTTCTCTCGTCCAACCT

81 G G G M L S I E L A N L Q G S * * T K T

241 CGGTGGAGGAATGCTTTCAATAGAGTTGGCCAACTTGCAGGGGTCGTAGTAAACAAAAAC

241 250 260 270 280 290

241 GCCACCTCCTTACGAAAGTTATCTCAACCGGTTGAACGTCCCCAGCATCATTTGTTTTTG

101 A L K W T V S T K S D Q I F D K L R R P

301 AGCCCTGAAGTGGACAGTATCAACGAAATCTGATCAAATATTTGATAAGTTGCGAAGACC

301 310 320 330 340 350

301 TCGGGACTTCACCTGTCATAGTTGCTTTAGACTAGTTTATAAACTATTCAACGCTTCTGG

121 M L V G P N R S N Y L V D M R S K I R D

361 AATGTTAGTAGGCCCTAATCGATCGAATTACTTAGTTGATATGCGAAGTAAGATAAGGGA

361 370 380 390 400 410

361 TTACAATCATCCGGGATTAGCTAGCTTAATGAATCAACTATACGCTTCATTCTATTCCCT

141 I N K L L D F E S D E V R F I G I V G M

421 TATCAATAAGCTACTTGACTTTGAATCAGATGAAGTACGATTTATAGGAATAGTTGGAAT

421 430 440 450 460 470

421 ATAGTTATTCGATGAACTGAAACTTAGTCTACTTCATGCTAAATATCCTTATCAACCTTA

161 G G I G K T T I A E V L H D S I A F T L

481 GGGTGGTATTGGTAAAACAACTATTGCAGAAGTTTTACACGACAGTATTGCATTTACATT

481 490 500 510 520 530

481 CCCACCATAACCATTTTGTTGATAACGTCTTCAAAATGTGCTGTCATAACGTAAATGTAA

181 F G E N S C F V T M S G R D I V T V Q C

541 GTTTGGCGAAAATTCTTGCTTTGTCACTATGTCTGGACGTGATATCGTCACGGTCCAATG

541 550 560 570 580 590

541 CAAACCGCTTTTAAGAACGAAACAGTGATACAGACCTGCACTATAGCAGTGCCAGGTTAC

201 L L L S R L L G T R E N I N I L E K N E

601 TCTACTACTCTCTCGACTTCTTGGAACTAGGGAGAATATTAACATTTTAGAAAAGAATGA

601 610 620 630 640 650

601 AGATGATGAGAGAGCTGAAGAACCTTGATCCCTCTTATAATTGTAAAATCTTTTCTTACT

221 G A N M I K D C L S G R K V L I I F D G

661 AGGAGCAAACATGATTAAAGATTGTTTGAGTGGGAGAAAGGTTTTGATTATTTTTGATGG

661 670 680 690 700 710

661 TCCTCGTTTGTACTAATTTCTAACAAACTCACCCTCTTTCCAAAACTAATAAAAACTACC

241 V N D R E E L G Y L A G S F D W F G R G

721 GGTGAATGATAGAGAGGAATTAGGATACTTAGCCGGAAGTTTTGATTGGTTTGGTCGAGG

721 730 740 750 760 770

721 CCACTTACTATCTCTCCTTAATCCTATGAATCGGCCTTCAAAACTAACCAAACCAGCTCC

261 S R V I I T T R N K N V F S H P N H E Q

781 AAGTCGAGTCATCATTACCACTAGAAATAAAAATGTTTTTTCTCACCCCAATCATGAACA

781 790 800 810 820 830

781 TTCAGCTCAGTAGTAATGGTGATCTTTATTTTTACAAAAAAGAGTGGGGTTAGTACTTGT

281 V Q L Y N V K P L D Y N T S F S L F W K

841 AGTTCAACTCTACAATGTGAAACCACTTGATTACAACACTTCATTCTCACTTTTTTGGAA

841 850 860 870 880 890

841 TCAAGTTGAGATGTTACACTTTGGTGAACTAATGTTGTGAAGTAAGAGTGAAAAAACCTT

301 H A F D Q Q S G G P S E Q Q F I Q L S Q

901 GCATGCATTTGATCAACAAAGTGGGGGTCCAAGTGAACAACAATTCATACAACTTAGTCA

901 910 920 930 940 950

901 CGTACGTAAACTAGTTGTTTCACCCCCAGGTTCACTTGTTGTTAAGTATGTTGAATCAGT

321 N I V E K V E G N P Q A L E Q I G S F L

961 GAATATAGTGGAAAAGGTCGAAGGAAATCCACAAGCATTGGAACAAATTGGATCATTTTT

961 970 980 990 1000 1010

961 CTTATATCACCTTTTCCAGCTTCCTTTAGGTGTTCGTAACCTTGTTTAACCTAGTAAAAA

341 R G K D I N V W K E E L K S L V L V D N

1021 GCGTGGTAAAGATATTAATGTATGGAAAGAAGAATTGAAGAGCCTTGTTTTAGTTGATAA

1021 1030 1040 1050 1060 1070

1021 CGCACCATTTCTATAATTACATACCTTTCTTCTTAACTTCTCGGAACAAAATCAACTATT

361 E R L F K I L K I S F D Q L G T K G Q Q

1081 TGAACGTCTCTTCAAAATATTAAAGATAAGTTTTGATCAATTAGGGACAAAAGGCCAACA

1081 1090 1100 1110 1120 1130

1081 ACTTGCAGAGAAGTTTTATAATTTCTATTCAAAACTAGTTAATCCCTGTTTTCCGGTTGT

381 A F L D L A C F F N G K S T G K I I E I

1141 AGCTTTTCTTGATTTGGCATGTTTCTTCAATGGAAAAAGTACAGGCAAAATTATTGAAAT

1141 1150 1160 1170 1180 1190

1141 TCGAAAAGAACTAAACCGTACAAAGAAGTTACCTTTTTCATGTCCGTTTTAATAACTTTA

401 L A S L E Y N S P S E V L K L L C D R Y

1201 ACTTGCGAGTTTAGAATACAATTCCCCCAGCGAAGTACTAAAGTTGTTGTGTGATAGATA

1201 1210 1220 1230 1240 1250

1201 TGAACGCTCAAATCTTATGTTAAGGGGGTCGCTTCATGATTTCAACAACACACTATCTAT

421 L I E I R D G D T V C M P N L I Q E M G

1261 TCTTATTGAAATTAGAGATGGAGACACAGTATGTATGCCCAATTTGATACAAGAAATGGG

1261 1270 1280 1290 1300 1310

1261 AGAATAACTTTAATCTCTACCTCTGTGTCATACATACGGGTTAAACTATGTTCTTTACCC

441 R E M D R Q E R Q R S R I W I R R D A F

1321 TCGAGAAATGGACCGACAAGAACGTCAAAGAAGCAGGATTTGGATTAGAAGAGATGCCTT

1321 1330 1340 1350 1360 1370

1321 AGCTCTTTACCTGGCTGTTCTTGCAGTTTCTTCGTCCTAAACCTAATCTTCTCTACGGAA

461 D I F D E Q H G V K D I K G V V L D K R

1381 CGACATATTTGATGAACAACATGGAGTAAAAGACATAAAAGGTGTTGTGTTGGACAAGAG

1381 1390 1400 1410 1420 1430

1381 GCTGTATAAACTACTTGTTGTACCTCATTTTCTGTATTTTCCACAACACAACCTGTTCTC

481 D T E P N L K L K A K Q L Q N M S R L K

1441 AGACACAGAACCAAACTTAAAGTTGAAGGCTAAACAATTACAAAATATGAGCCGTTTAAA

1441 1450 1460 1470 1480 1490

1441 TCTGTGTCTTGGTTTGAATTTCAACTTCCGATTTGTTAATGTTTTATACTCGGCAAATTT

501 I L E I D N V Q L S P R N Q N D L S N Q

1501 AATATTAGAGATTGACAATGTGCAGCTTAGTCCAAGAAATCAAAATGATCTCTCTAATCA

1501 1510 1520 1530 1540 1550

1501 TTATAATCTCTAACTGTTACACGTCGAATCAGGTTCTTTAGTTTTACTAGAGAGATTAGT

521 L R L L H W D G F P S D T L P L N F E A

1561 GCTTCGATTGCTTCACTGGGATGGCTTTCCTTCAGACACTTTGCCACTAAATTTTGAAGC

1561 1570 1580 1590 1600 1610

1561 CGAAGCTAACGAAGTGACCCTACCGAAAGGAAGTCTGTGAAACGGTGATTTAAAACTTCG

541 P Y L F E L L L P N A Q T T H L W K E L

1621 ACCATATTTATTTGAACTTCTCTTGCCTAATGCTCAAACCACTCATCTTTGGAAAGAACT

1621 1630 1640 1650 1660 1670

1621 TGGTATAAATAAACTTGAAGAGAACGGATTACGAGTTTGGTGAGTAGAAACCTTTCTTGA

561 K G F K K L K V I D V S N S R T L V E T

1681 AAAGGGATTTAAGAAATTAAAGGTAATCGATGTTAGCAATTCCCGAACTTTGGTGGAGAC

1681 1690 1700 1710 1720 1730

1681 TTTCCCTAAATTCTTTAATTTCCATTAGCTACAATCGTTAAGGGCTTGAAACCACCTCTG

581 P N L S A V P N L E R L I L C N C T R L

1741 ACCGAATTTAAGTGCTGTTCCAAATCTAGAAAGATTGATTCTATGTAATTGTACAAGATT

1741 1750 1760 1770 1780 1790

1741 TGGCTTAAATTCACGACAAGGTTTAGATCTTTCTAACTAAGATACATTAACATGTTCTAA

601 K K I D N S I T K L R L L V L V D L T G

1801 GAAGAAAATTGATAATTCAATTACAAAATTGAGACTTCTAGTTTTAGTAGACCTCACAGG

1801 1810 1820 1830 1840 1850

1801 CTTCTTTTAACTATTAAGTTAATGTTTTAACTCTGAAGATCAAAATCATCTGGAGTGTCC

621 C V R L E T S E C I D I L K S R P T V E

1861 CTGTGTTCGCCTCGAAACATCCGAGTGCATCGATATCTTAAAGAGTCGCCCAACAGTAGA

1861 1870 1880 1890 1900 1910

1861 GACACAAGCGGAGCTTTGTAGGCTCACGTAGCTATAGAATTTCTCAGCGGGTTGTCATCT

641 L R G L V L Q C H Q S P I I R R Y L L Y

1921 ACTTCGTGGCTTAGTTCTACAGTGTCACCAATCACCAATCATTAGAAGGTATTTGCTATA

1921 1930 1940 1950 1960 1970

1921 TGAAGCACCGAATCAAGATGTCACAGTGGTTAGTGGTTAGTAATCTTCCATAAACGATAT

661 L * I L S L I F * N L G S S R V

1981 TTTGTAAATTCTTTCGCTTATCTTTTAAAATTTAGGATCATCTCGTGTAA

1981 1990 2000 2010 2020

1981 AAACATTTAAGAAAGCGAATAGAAAATTTTAAATCCTAGTAGAGCACATT

**b) Cornel ZPPM 339**


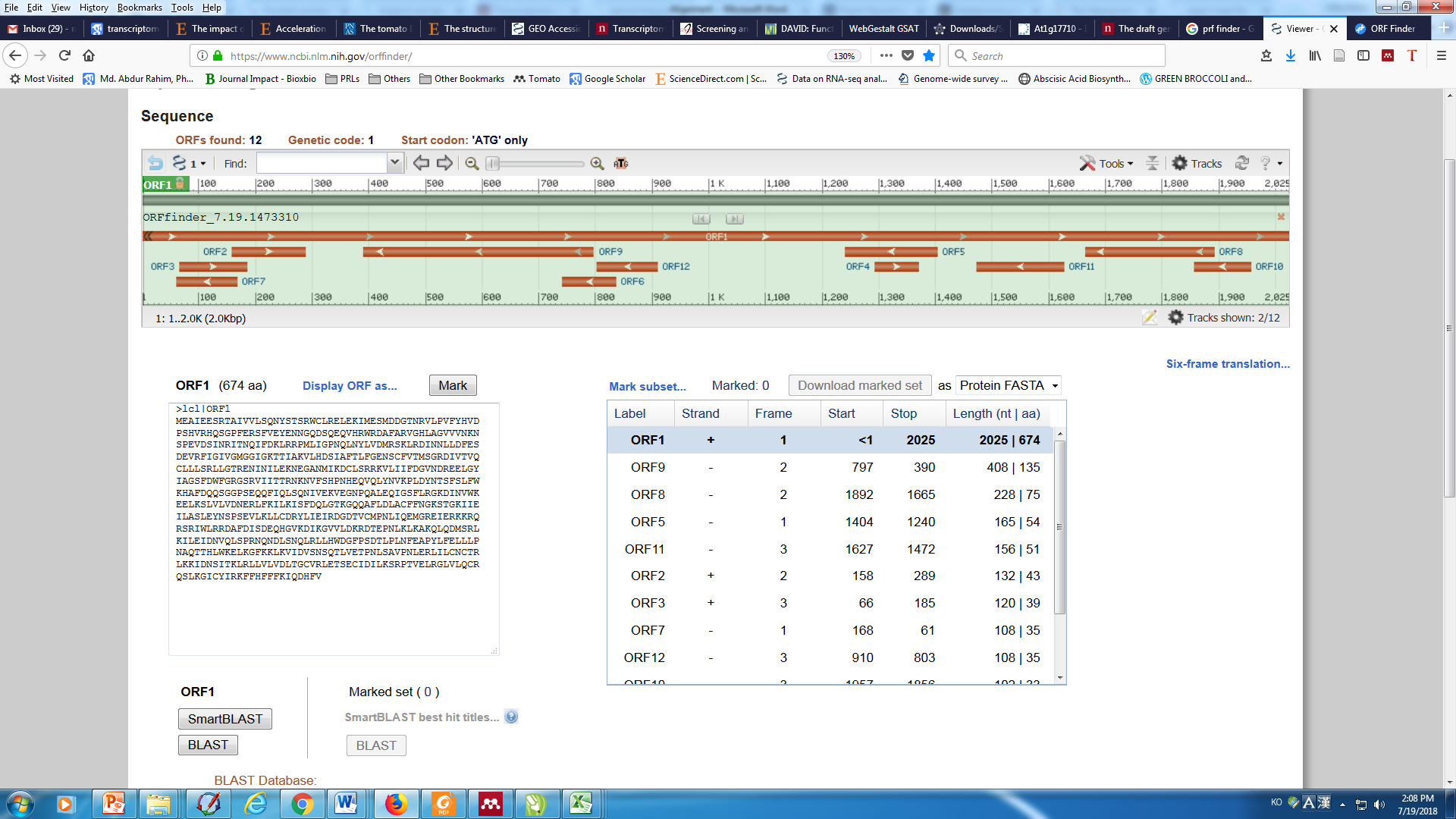


1 M E A I E E S R T A I V V L S Q N Y S T

1 ATGGAAGCAATTGAGGAATCAAGAACAGCTATTGTGGTTTTATCACAAAACTATTCTACT

1 10 20 30 40 50

1 TACCTTCGTTAACTCCTTAGTTCTTGTCGATAACACCAAAATAGTGTTTTGATAAGATGA

21 S R W C L R E L E K I M E S M D D G T N

61 TCAAGATGGTGCTTGAGAGAATTGGAGAAGATTATGGAATCCATGGACGACGGAACAAAT

61 70 80 90 100 110

61 AGTTCTACCACGAACTCTCTTAACCTCTTCTAATACCTTAGGTACCTGCTGCCTTGTTTA

41 R V L P V F Y H V D P S H V R H Q S G P

121 CGAGTTCTTCCTGTGTTTTACCATGTAGATCCTTCTCATGTTCGTCATCAATCTGGACCT

121 130 140 150 160 170

121 GCTCAAGAAGGACACAAAATGGTACATCTAGGAAGAGTACAAGCAGTAGTTAGACCTGGA

61 F E R S F V E Y E N N G Q D S Q E Q V H

181 TTTGAGAGAAGCTTTGTTGAATATGAAAATAATGGACAAGACTCACAAGAGCAGGTTCAT

181 190 200 210 220 230

181 AAACTCTCTTCGAAACAACTTATACTTTTATTACCTGTTCTGAGTGTTCTCGTCCAAGTA

81 R W R D A F A R V G H L A G V V V N K N

241 CGGTGGAGGGATGCTTTCGCTAGAGTTGGCCATCTTGCAGGGGTCGTAGTAAACAAAAAC

241 250 260 270 280 290

241 GCCACCTCCCTACGAAAGCGATCTCAACCGGTAGAACGTCCCCAGCATCATTTGTTTTTG

101 S P E V D S I N R I T N Q I F D K L R R

301 AGCCCTGAAGTGGACAGTATCAACCGAATCACCAATCAAATATTTGATAAGTTGCGAAGA

301 310 320 330 340 350

301 TCGGGACTTCACCTGTCATAGTTGGCTTAGTGGTTAGTTTATAAACTATTCAACGCTTCT

121 P M L I G P N Q L N Y L V D M R S K L R

361 CCTATGTTAATAGGCCCTAATCAATTGAATTACTTGGTTGATATGCGAAGTAAGCTAAGG

361 370 380 390 400 410

361 GGATACAATTATCCGGGATTAGTTAACTTAATGAACCAACTATACGCTTCATTCGATTCC

141 D I N N L L D F E S D E V R F I G I V G

421 GATATCAATAACCTACTTGACTTTGAATCAGATGAAGTACGATTTATAGGAATAGTTGGA

421 430 440 450 460 470

421 CTATAGTTATTGGATGAACTGAAACTTAGTCTACTTCATGCTAAATATCCTTATCAACCT

161 M G G I G K T T I A K V L H D S I A F T

481 ATGGGTGGTATTGGTAAAACAACTATTGCAAAAGTTTTACACGACAGTATTGCATTTACA

481 490 500 510 520 530

481 TACCCACCATAACCATTTTGTTGATAACGTTTTCAAAATGTGCTGTCATAACGTAAATGT

181 L F G E N S C F V T M S G R D I V T V Q

541 TTATTTGGTGAAAATTCTTGCTTTGTCACTATGTCTGGGCGTGATATCGTCACGGTCCAA

541 550 560 570 580 590

541 AATAAACCACTTTTAAGAACGAAACAGTGATACAGACCCGCACTATAGCAGTGCCAGGTT

201 C L L L S R L L G T R E N I N I L E K N

601 TGTCTACTACTCTCTCGACTTCTTGGAACTAGGGAGAATATTAACATTTTAGAAAAGAAT

601 610 620 630 640 650

601 ACAGATGATGAGAGAGCTGAAGAACCTTGATCCCTCTTATAATTGTAAAATCTTTTCTTA

221 E G A N M I K D C L S R R K V L I I F D

661 GAAGGAGCAAACATGATTAAAGATTGTTTGAGTAGGAGAAAGGTTTTGATTATTTTTGAT

661 670 680 690 700 710

661 CTTCCTCGTTTGTACTAATTTCTAACAAACTCATCCTCTTTCCAAAACTAATAAAAACTA

241 G V N D R E E L G Y I A G S F D W F G R

721 GGGGTGAATGATAGAGAGGAATTAGGATACATAGCCGGAAGTTTTGATTGGTTTGGTCGA

721 730 740 750 760 770

721 CCCCACTTACTATCTCTCCTTAATCCTATGTATCGGCCTTCAAAACTAACCAAACCAGCT

261 G S R V I I T T R N K N V F S H P N H E

781 GGAAGTCGAGTCATCATTACCACTAGAAATAAAAATGTTTTTTCTCACCCCAATCATGAA

781 790 800 810 820 830

781 CCTTCAGCTCAGTAGTAATGGTGATCTTTATTTTTACAAAAAAGAGTGGGGTTAGTACTT

281 Q V Q L Y N V K P L D Y N T S F S L F W

841 CAAGTTCAACTCTACAATGTGAAACCACTTGATTACAACACTTCATTCTCACTTTTTTGG

841 850 860 870 880 890

841 GTTCAAGTTGAGATGTTACACTTTGGTGAACTAATGTTGTGAAGTAAGAGTGAAAAAACC

301 K H A F D Q Q S G G P S E Q Q F I Q L S

901 AAGCATGCATTTGATCAACAAAGTGGGGGTCCAAGTGAACAACAATTCATACAACTTAGT

901 910 920 930 940 950

901 TTCGTACGTAAACTAGTTGTTTCACCCCCAGGTTCACTTGTTGTTAAGTATGTTGAATCA

321 Q N I V E K V E G N P Q A L E Q I G S F

961 CAGAATATAGTGGAAAAGGTCGAAGGAAATCCACAAGCATTGGAACAAATTGGATCATTT

961 970 980 990 1000 1010

961 GTCTTATATCACCTTTTCCAGCTTCCTTTAGGTGTTCGTAACCTTGTTTAACCTAGTAAA

341 L R G K D I N V W K E E L K S L V L V D

1021 TTGCGTGGTAAAGATATTAATGTATGGAAAGAAGAATTGAAGAGCCTTGTTTTAGTTGAT

1021 1030 1040 1050 1060 1070

1021 AACGCACCATTTCTATAATTACATACCTTTCTTCTTAACTTCTCGGAACAAAATCAACTA

361 N E R L F K I L K I S F D Q L G T K G Q

1081 AATGAACGTCTCTTCAAAATATTAAAGATAAGTTTTGATCAATTAGGGACAAAAGGCCAA

1081 1090 1100 1110 1120 1130

1081 TTACTTGCAGAGAAGTTTTATAATTTCTATTCAAAACTAGTTAATCCCTGTTTTCCGGTT

381 Q A F L D L A C F F N G K S T G K I I E

1141 CAAGCTTTTCTTGATTTGGCATGTTTCTTCAATGGAAAAAGTACAGGCAAAATTATTGAA

1141 1150 1160 1170 1180 1190

1141 GTTCGAAAAGAACTAAACCGTACAAAGAAGTTACCTTTTTCATGTCCGTTTTAATAACTT

401 I L A S L E Y N S P S E V L K L L C D R

1201 ATACTTGCGAGTTTAGAATACAATTCCCCCAGCGAAGTACTAAAGTTGTTGTGTGATAGA

1201 1210 1220 1230 1240 1250

1201 TATGAACGCTCAAATCTTATGTTAAGGGGGTCGCTTCATGATTTCAACAACACACTATCT

421 Y L I E I R D G D T V C M P N L I Q E M

1261 TATCTTATTGAAATTAGAGATGGAGACACAGTATGTATGCCCAATTTGATACAAGAAATG

1261 1270 1280 1290 1300 1310

1261 ATAGAATAACTTTAATCTCTACCTCTGTGTCATACATACGGGTTAAACTATGTTCTTTAC

441 G R E I E R K K R Q R S R I W L R R D A

1321 GGTCGAGAAATAGAACGAAAAAAACGTCAAAGAAGCAGGATTTGGCTTAGAAGAGATGCT

1321 1330 1340 1350 1360 1370

1321 CCAGCTCTTTATCTTGCTTTTTTTGCAGTTTCTTCGTCCTAAACCGAATCTTCTCTACGA

461 F D I S D E Q H G V K D I K G V V L D K

1381 TTCGACATATCTGATGAACAACATGGAGTAAAAGACATAAAAGGTGTTGTGTTGGACAAG

1381 1390 1400 1410 1420 1430

1381 AAGCTGTATAGACTACTTGTTGTACCTCATTTTCTGTATTTTCCACAACACAACCTGTTC

481 R D T E P N L K L K A K Q L Q D M S R L

1441 AGAGACACAGAACCAAACTTAAAGTTGAAGGCTAAACAATTACAAGATATGAGCCGTTTA

1441 1450 1460 1470 1480 1490

1441 TCTCTGTGTCTTGGTTTGAATTTCAACTTCCGATTTGTTAATGTTCTATACTCGGCAAAT

501 K I L E I D N V Q L S P R N Q N D L S N

1501 AAAATATTAGAGATTGACAATGTGCAGCTGAGTCCAAGAAATCAAAATGATCTCTCAAAT

1501 1510 1520 1530 1540 1550

1501 TTTTATAATCTCTAACTGTTACACGTCGACTCAGGTTCTTTAGTTTTACTAGAGAGTTTA

521 Q L R L L H W D G F P S D T L P L N F E

1561 CAGCTTCGATTGCTCCACTGGGATGGCTTTCCTTCAGACACTTTGCCACTAAATTTCGAA

1561 1570 1580 1590 1600 1610

1561 GTCGAAGCTAACGAGGTGACCCTACCGAAAGGAAGTCTGTGAAACGGTGATTTAAAGCTT

541 A P Y L F E L L L P N A Q T T H L W K E

1621 GCACCATATTTATTTGAACTTCTCTTGCCTAATGCACAAACCACTCATCTTTGGAAAGAA

1621 1630 1640 1650 1660 1670

1621 CGTGGTATAAATAAACTTGAAGAGAACGGATTACGTGTTTGGTGAGTAGAAACCTTTCTT

561 L K G F K K L K V I D V S N S Q T L V E

1681 CTAAAGGGATTTAAGAAATTAAAGGTAATCGATGTTAGCAATTCCCAAACTTTGGTGGAG

1681 1690 1700 1710 1720 1730

1681 GATTTCCCTAAATTCTTTAATTTCCATTAGCTACAATCGTTAAGGGTTTGAAACCACCTC

581 T P N L S A V P N L E R L I L C N C T R

1741 ACACCGAATTTGAGTGCTGTTCCAAATCTAGAAAGATTGATTCTATGTAATTGTACAAGA

1741 1750 1760 1770 1780 1790

1741 TGTGGCTTAAACTCACGACAAGGTTTAGATCTTTCTAACTAAGATACATTAACATGTTCT

601 L K K I D N S I T K L R L L V L V D L T

1801 TTGAAGAAAATTGATAATTCAATTACAAAATTGAGACTTCTAGTTTTAGTAGACCTCACA

1801 1810 1820 1830 1840 1850

1801 AACTTCTTTTAACTATTAAGTTAATGTTTTAACTCTGAAGATCAAAATCATCTGGAGTGT

621 G C V R L E T S E C I D I L K S R P T V

1861 GGCTGTGTTCGCCTCGAAACATCCGAGTGCATCGATATCTTGAAGAGTCGCCCAACAGTA

1861 1870 1880 1890 1900 1910

1861 CCGACACAAGCGGAGCTTTGTAGGCTCACGTAGCTATAGAACTTCTCAGCGGGTTGTCAT

641 E L R G L V L Q C R Q S L K G I C Y I R

1921 GAACTTCGTGGCTTAGTTCTACAGTGTCGCCAATCATTGAAAGGTATTTGTTATATTCGT

1921 1930 1940 1950 1960 1970

1921 CTTGAAGCACCGAATCAAGATGTCACAGCGGTTAGTAACTTTCCATAAACAATATAAGCA

661 K F F H F F F K I Q D H F V *

1981 AAATTTTTTCACTTCTTTTTTAAAATTCAGGATCATTTTGTGTAA

1981 1990 2000 2010 2020

1981 TTTAAAAAAGTGAAGAAAAAATTTTAAGTCCTAGTAAAACACATT

**C. Coding sequence (CDS) and protein sequence**

>MELO3C022157T1 (CDS) Reference

ATGGAAGCAATTGAGGAATCAAGAACAGCTATTGTGGTTTTATCACAAAACTATTCTACTTCAAGATGGTGCTTGAGAGAATTGGAGAAGATTATGGAATCCATGGACGACGGAACAAATCGAGTTCTTCCTGTGTTTTACCATGTAGATCCTTCTCATGTTCGTCATCAATCTGGACCTTTTGAGAGAAGCTTTGTTGAATATGAAAATAATGGACAAGACTCACAAGAGCAGGTTCATCGGTGGAGGGATGCTTTCGCTAGAGTTGGCCATCTTGCAGGGGTCGTAGTAAACAAAAACAGCCCTGAAGTGGACAGTATCAACCGAATCACCAATCAAATATTTGATAAGTTGCGAAGACCTATGTTAATAGGCCCTAATCAATTGAATTACTTGGTTGATATGCGAAGTAAGCTAAGGGATATCAATAACCTACTTGACTTTGAATCAGATGAAGTACGATTTATAGGAATAGTTGGAATGGGTGGTATTGGTAAAACAACTATTGCAAAAGTTTTACACGACAGTATTGCATTTACATTATTTGGTGAAAATTCTTGCTTTGTCACTATGTCTGGGCGTGATATCGTCACGGTCCAATGTCTACTACTCTCTCGACTTCTTGGAACTAGGGAGAATATTAACATTTTAGAAAAGAATGAAGGAGCAAACATGATTAAAGATTGTTTGAGTAGGAGAAAGGTTTTGATTATTTTTGATGGGGTGAATGATAGAGAGGAATTAGGATACATAGCCGGAAGTTTTGATTGGTTTGGTCGAGGAAGTCGAGTCATCATTACCACTAGAAATAAAAATGTTTTTTCTCACCCCAATCATGAACAAGTTCAACTCTACAATGTGAAACCACTTGATTACAACACTTCATTCTCACTTTTTTGGAAGCATGCATTTGATCAACAAAGTGGGGGTCCAAGTGAACAACAATTCATACAACTTAGTCAGAATATAGTGGAAAAGGTCGAAGGAAATCCACAAGCATTGGAACAAATTGGATCATTTTTGCGTGGTAAAGATATTAATGTATGGAAAGAAGAATTGAAGAGCCTTGTTTTAGTTGATAATGAACGTCTCTTCAAAATATTAAAGATAAGTTTTGATCAATTAGGGACAAAAGGCCAACAAGCTTTTCTTGATTTGGCATGTTTCTTCAATGGAAAAAGTACAGGCAAAATTATTGAAATACTTGCGAGTTTAGAATACAATTCCCCCAGCGAAGTACTAAAGTTGTTGTGTGATAGATATCTTATTGAAATTAGAGATGGAGACACAGTATGTATGCCCAATTTGATACAAGAAATGGGTCGAGAAATAGAACGAAAAAAACGTCAAAGAAGCAGGATTTGGCTTAGAAGAGATGCTTTCGACATATTTGATGAACAACATGGAGTAAAAGACATAAAAGGTGTTGTGTTGGACAAGAGAGACACAGAACCAAACTTAAAGTTGAAGGCTAAACAATTACAAGATATGAGCCGTTTAAAAATATTAGAGATTGACAATGTGCAGCTGAGTCCAAGAAATCAAAATGATCTCTCAAATCAGCTTCGATTGCTCCACTGGGATGGCTTTCCTTCAGACACTTTGCCACTAAATTTCGAAGCACCATATTTATTTGAACTTCTCTTGCCTAATGCACAAACCACTCATCTTTGGAAAGAACTAAAGGGATTTAAGAAATTAAAGGTAATCGATGTTAGCAATTCCCAAACTTTGGTGGAGACACCGAATTTGAGTGCTGTTCCAAATCTAGAAAGATTGATTCTATGTAATTGTACAAGATTGAAGAAAATTGATAATTCAATTACAAAATTGAGACTTCTAGTTTTAGTAGACCTCACAGGCTGTGTTCGCCTCGAAACATCCGAGTGCATCGATATCTTGAAGAGTCGCCCAACAGTAGAACTTCGTGGCTTAGTTCTACAGTGTCGCCAATCATTGAAAGGTATTTGTTATATTCGTAAATTTTTTCACTTCTTTTTTAAAATTCAGGATCATTTTGTGTAA

>PI482399 (CDS) Resistant line

ATGGAAGCAATTGAGGAATCAAGAACAGCTATTGTGGTTTTATCACAAAACTATTCTACTTCAAGATGGTGCTTGAGAGAGTTGGAGAAGATTATGGAATCCATGGACGACGGAACAAATCGAGTTCTTCCTGTGTTTTACCATGTAGATCCTTCTCATGTTCGTCATCAATCTGGACCTTTTGAGAGAAGCTTTGTTGAATATGAAAATAATAGACAATACTCAAGAGAGCAGGTTGGACGGTGGAGGAATGCTTTCAATAGAGTTGGCCAACTTGCAGGGGTCGTAGTAAACAAAAACAGCCCTGAAGTGGACAGTATCAACGAAATCTGATCAAATATTTGATAAGTTGCGAAGACCAATGTTAGTAGGCCCTAATCGATCGAATTACTTAGTTGATATGCGAAGTAAGATAAGGGATATCAATAAGCTACTTGACTTTGAATCAGATGAAGTACGATTTATAGGAATAGTTGGAATGGGTGGTATTGGTAAAACAACTATTGCAGAAGTTTTACACGACAGTATTGCATTTACATTGTTTGGCGAAAATTCTTGCTTTGTCACTATGTCTGGACGTGATATCGTCACGGTCCAATGTCTACTACTCTCTCGACTTCTTGGAACTAGGGAGAATATTAACATTTTAGAAAAGAATGAAGGAGCAAACATGATTAAAGATTGTTTGAGTGGGAGAAAGGTTTTGATTATTTTTGATGGGGTGAATGATAGAGAGGAATTAGGATACTTAGCCGGAAGTTTTGATTGGTTTGGTCGAGGAAGTCGAGTCATCATTACCACTAGAAATAAAAATGTTTTTTCTCACCCCAATCATGAACAAGTTCAACTCTACAATGTGAAACCACTTGATTACAACACTTCATTCTCACTTTTTTGGAAGCATGCATTTGATCAACAAAGTGGGGGTCCAAGTGAACAACAATTCATACAACTTAGTCAGAATATAGTGGAAAAGGTCGAAGGAAATCCACAAGCATTGGAACAAATTGGATCATTTTTGCGTGGTAAAGATATTAATGTATGGAAAGAAGAATTGAAGAGCCTTGTTTTAGTTGATAATGAACGTCTCTTCAAAATATTAAAGATAAGTTTTGATCAATTAGGGACAAAAGGCCAACAAGCTTTTCTTGATTTGGCATGTTTCTTCAATGGAAAAAGTACAGGCAAAATTATTGAAATACTTGCGAGTTTAGAATACAATTCCCCCAGCGAAGTACTAAAGTTGTTGTGTGATAGATATCTTATTGAAATTAGAGATGGAGACACAGTATGTATGCCCAATTTGATACAAGAAATGGGTCGAGAAATGGACCGACAAGAACGTCAAAGAAGCAGGATTTGGATTAGAAGAGATGCCTTCGACATATTTGATGAACAACATGGAGTAAAAGACATAAAAGGTGTTGTGTTGGACAAGAGAGACACAGAACCAAACTTAAAGTTGAAGGCTAAACAATTACAAAATATGAGCCGTTTAAAAATATTAGAGATTGACAATGTGCAGCTTAGTCCAAGAAATCAAAATGATCTCTCTAATCAGCTTCGATTGCTTCACTGGGATGGCTTTCCTTCAGACACTTTGCCACTAAATTTTGAAGCACCATATTTATTTGAACTTCTCTTGCCTAATGCTCAAACCACTCATCTTTGGAAAGAACTAAAGGGATTTAAGAAATTAAAGGTAATCGATGTTAGCAATTCCCGAACTTTGGTGGAGACACCGAATTTAAGTGCTGTTCCAAATCTAGAAAGATTGATTCTATGTAATTGTACAAGATTGAAGAAAATTGATAATTCAATTACAAAATTGAGACTTCTAGTTTTAGTAGACCTCACAGGCTGTGTTCGCCTCGAAACATCCGAGTGCATCGATATCTTAAAGAGTCGCCCAACAGTAGAACTTCGTGGCTTAGTTCTACAGTGTCACCAATCACCAATCATTAGAAGGTATTTGCTATATTTGTAAATTCTTTCGCTTATCTTTTAAAATTTAGGATCATCTCGTGTAA

>Cornel ZPPM 339 (CDS) Susceptible line

ATGGAAGCAATTGAGGAATCAAGAACAGCTATTGTGGTTTTATCACAAAACTATTCTACTTCAAGATGGTGCTTGAGAGAATTGGAGAAGATTATGGAATCCATGGACGACGGAACAAATCGAGTTCTTCCTGTGTTTTACCATGTAGATCCTTCTCATGTTCGTCATCAATCTGGACCTTTTGAGAGAAGCTTTGTTGAATATGAAAATAATGGACAAGACTCACAAGAGCAGGTTCATCGGTGGAGGGATGCTTTCGCTAGAGTTGGCCATCTTGCAGGGGTCGTAGTAAACAAAAACAGCCCTGAAGTGGACAGTATCAACCGAATCACCAATCAAATATTTGATAAGTTGCGAAGACCTATGTTAATAGGCCCTAATCAATTGAATTACTTGGTTGATATGCGAAGTAAGCTAAGGGATATCAATAACCTACTTGACTTTGAATCAGATGAAGTACGATTTATAGGAATAGTTGGAATGGGTGGTATTGGTAAAACAACTATTGCAAAAGTTTTACACGACAGTATTGCATTTACATTATTTGGTGAAAATTCTTGCTTTGTCACTATGTCTGGGCGTGATATCGTCACGGTCCAATGTCTACTACTCTCTCGACTTCTTGGAACTAGGGAGAATATTAACATTTTAGAAAAGAATGAAGGAGCAAACATGATTAAAGATTGTTTGAGTAGGAGAAAGGTTTTGATTATTTTTGATGGGGTGAATGATAGAGAGGAATTAGGATACATAGCCGGAAGTTTTGATTGGTTTGGTCGAGGAAGTCGAGTCATCATTACCACTAGAAATAAAAATGTTTTTTCTCACCCCAATCATGAACAAGTTCAACTCTACAATGTGAAACCACTTGATTACAACACTTCATTCTCACTTTTTTGGAAGCATGCATTTGATCAACAAAGTGGGGGTCCAAGTGAACAACAATTCATACAACTTAGTCAGAATATAGTGGAAAAGGTCGAAGGAAATCCACAAGCATTGGAACAAATTGGATCATTTTTGCGTGGTAAAGATATTAATGTATGGAAAGAAGAATTGAAGAGCCTTGTTTTAGTTGATAATGAACGTCTCTTCAAAATATTAAAGATAAGTTTTGATCAATTAGGGACAAAAGGCCAACAAGCTTTTCTTGATTTGGCATGTTTCTTCAATGGAAAAAGTACAGGCAAAATTATTGAAATACTTGCGAGTTTAGAATACAATTCCCCCAGCGAAGTACTAAAGTTGTTGTGTGATAGATATCTTATTGAAATTAGAGATGGAGACACAGTATGTATGCCCAATTTGATACAAGAAATGGGTCGAGAAATAGAACGAAAAAAACGTCAAAGAAGCAGGATTTGGCTTAGAAGAGATGCTTTCGACATATCTGATGAACAACATGGAGTAAAAGACATAAAAGGTGTTGTGTTGGACAAGAGAGACACAGAACCAAACTTAAAGTTGAAGGCTAAACAATTACAAGATATGAGCCGTTTAAAAATATTAGAGATTGACAATGTGCAGCTGAGTCCAAGAAATCAAAATGATCTCTCAAATCAGCTTCGATTGCTCCACTGGGATGGCTTTCCTTCAGACACTTTGCCACTAAATTTCGAAGCACCATATTTATTTGAACTTCTCTTGCCTAATGCACAAACCACTCATCTTTGGAAAGAACTAAAGGGATTTAAGAAATTAAAGGTAATCGATGTTAGCAATTCCCAAACTTTGGTGGAGACACCGAATTTGAGTGCTGTTCCAAATCTAGAAAGATTGATTCTATGTAATTGTACAAGATTGAAGAAAATTGATAATTCAATTACAAAATTGAGACTTCTAGTTTTAGTAGACCTCACAGGCTGTGTTCGCCTCGAAACATCCGAGTGCATCGATATCTTGAAGAGTCGCCCAACAGTAGAACTTCGTGGCTTAGTTCTACAGTGTCGCCAATCATTGAAAGGTATTTGTTATATTCGTAAATTTTTTCACTTCTTTTTTAAAATTCAGGATCATTTTGTGTAA

**Coding sequence (CDS) alignment**

MELO3C022157T1 ATGGAAGCAATTGAGGAATCAAGAACAGCTATTGTGGTTTTATCACAAAACTATTCTACT 60

PI482399 ATGGAAGCAATTGAGGAATCAAGAACAGCTATTGTGGTTTTATCACAAAACTATTCTACT 60

Cornel ATGGAAGCAATTGAGGAATCAAGAACAGCTATTGTGGTTTTATCACAAAACTATTCTACT 60

************************************************************

MELO3C022157T1 TCAAGATGGTGCTTGAGAGAATTGGAGAAGATTATGGAATCCATGGACGACGGAACAAAT 120

PI482399 TCAAGATGGTGCTTGAGAGAGTTGGAGAAGATTATGGAATCCATGGACGACGGAACAAAT 120

Cornel TCAAGATGGTGCTTGAGAGAATTGGAGAAGATTATGGAATCCATGGACGACGGAACAAAT 120

********************.***************************************

MELO3C022157T1 CGAGTTCTTCCTGTGTTTTACCATGTAGATCCTTCTCATGTTCGTCATCAATCTGGACCT 180

PI482399 CGAGTTCTTCCTGTGTTTTACCATGTAGATCCTTCTCATGTTCGTCATCAATCTGGACCT 180

Cornel CGAGTTCTTCCTGTGTTTTACCATGTAGATCCTTCTCATGTTCGTCATCAATCTGGACCT 180

************************************************************

MELO3C022157T1 TTTGAGAGAAGCTTTGTTGAATATGAAAATAATGGACAAGACTCACAAGAGCAGGTTCAT 240

PI482399 TTTGAGAGAAGCTTTGTTGAATATGAAAATAATAGACAATACTCAAGAGAGCAGGTTGGA 240

Cornel TTTGAGAGAAGCTTTGTTGAATATGAAAATAATGGACAAGACTCACAAGAGCAGGTTCAT 240

*********************************.***** *****..********** .:

MELO3C022157T1 CGGTGGAGGGATGCTTTCGCTAGAGTTGGCCATCTTGCAGGGGTCGTAGTAAACAAAAAC 300

PI482399 CGGTGGAGGAATGCTTTCAATAGAGTTGGCCAACTTGCAGGGGTCGTAGTAAACAAAAAC 300

Cornel CGGTGGAGGGATGCTTTCGCTAGAGTTGGCCATCTTGCAGGGGTCGTAGTAAACAAAAAC 300

*********.********..************:***************************

MELO3C022157T1 AGCCCTGAAGTGGACAGTATCAACCGAATCACCAATCAAATATTTGATAAGTTGCGAAGA 360

PI482399 AGCCCTGAAGTGGACAGTATCAACGAAATC--TGATCAAATATTTGATAAGTTGCGAAGA 358

Cornel AGCCCTGAAGTGGACAGTATCAACCGAATCACCAATCAAATATTTGATAAGTTGCGAAGA 360

************************ .**** .**************************

**2-bp deletion (2^nd^ exon)**

MELO3C022157T1 CCTATGTTAATAGGCCCTAATCAATTGAATTACTTGGTTGATATGCGAAGTAAGCTAAGG 420

PI482399 CCAATGTTAGTAGGCCCTAATCGATCGAATTACTTAGTTGATATGCGAAGTAAGATAAGG 418

Cornel CCTATGTTAATAGGCCCTAATCAATTGAATTACTTGGTTGATATGCGAAGTAAGCTAAGG 420

**:******.************.** *********.******************.*****

MELO3C022157T1 GATATCAATAACCTACTTGACTTTGAATCAGATGAAGTACGATTTATAGGAATAGTTGGA 480

PI482399 GATATCAATAAGCTACTTGACTTTGAATCAGATGAAGTACGATTTATAGGAATAGTTGGA 478

Cornel GATATCAATAACCTACTTGACTTTGAATCAGATGAAGTACGATTTATAGGAATAGTTGGA 480

*********** ************************************************

MELO3C022157T1 ATGGGTGGTATTGGTAAAACAACTATTGCAAAAGTTTTACACGACAGTATTGCATTTACA 540

PI482399 ATGGGTGGTATTGGTAAAACAACTATTGCAGAAGTTTTACACGACAGTATTGCATTTACA 538

Cornel ATGGGTGGTATTGGTAAAACAACTATTGCAAAAGTTTTACACGACAGTATTGCATTTACA 540

******************************.*****************************

MELO3C022157T1 TTATTTGGTGAAAATTCTTGCTTTGTCACTATGTCTGGGCGTGATATCGTCACGGTCCAA 600

PI482399 TTGTTTGGCGAAAATTCTTGCTTTGTCACTATGTCTGGACGTGATATCGTCACGGTCCAA 598

Cornel TTATTTGGTGAAAATTCTTGCTTTGTCACTATGTCTGGGCGTGATATCGTCACGGTCCAA 600

**.***** *****************************.*********************

MELO3C022157T1 TGTCTACTACTCTCTCGACTTCTTGGAACTAGGGAGAATATTAACATTTTAGAAAAGAAT 660

PI482399 TGTCTACTACTCTCTCGACTTCTTGGAACTAGGGAGAATATTAACATTTTAGAAAAGAAT 658

Cornel TGTCTACTACTCTCTCGACTTCTTGGAACTAGGGAGAATATTAACATTTTAGAAAAGAAT 660

************************************************************

MELO3C022157T1 GAAGGAGCAAACATGATTAAAGATTGTTTGAGTAGGAGAAAGGTTTTGATTATTTTTGAT 720

PI482399 GAAGGAGCAAACATGATTAAAGATTGTTTGAGTGGGAGAAAGGTTTTGATTATTTTTGAT 718

Cornel GAAGGAGCAAACATGATTAAAGATTGTTTGAGTAGGAGAAAGGTTTTGATTATTTTTGAT 720

*********************************.**************************

MELO3C022157T1 GGGGTGAATGATAGAGAGGAATTAGGATACATAGCCGGAAGTTTTGATTGGTTTGGTCGA 780

PI482399 GGGGTGAATGATAGAGAGGAATTAGGATACTTAGCCGGAAGTTTTGATTGGTTTGGTCGA 778

Cornel GGGGTGAATGATAGAGAGGAATTAGGATACATAGCCGGAAGTTTTGATTGGTTTGGTCGA 780

******************************:*****************************

MELO3C022157T1 GGAAGTCGAGTCATCATTACCACTAGAAATAAAAATGTTTTTTCTCACCCCAATCATGAA 840

PI482399 GGAAGTCGAGTCATCATTACCACTAGAAATAAAAATGTTTTTTCTCACCCCAATCATGAA 838

Cornel GGAAGTCGAGTCATCATTACCACTAGAAATAAAAATGTTTTTTCTCACCCCAATCATGAA 840

************************************************************

MELO3C022157T1 CAAGTTCAACTCTACAATGTGAAACCACTTGATTACAACACTTCATTCTCACTTTTTTGG 900

PI482399 CAAGTTCAACTCTACAATGTGAAACCACTTGATTACAACACTTCATTCTCACTTTTTTGG 898

Cornel CAAGTTCAACTCTACAATGTGAAACCACTTGATTACAACACTTCATTCTCACTTTTTTGG 900

************************************************************

MELO3C022157T1 AAGCATGCATTTGATCAACAAAGTGGGGGTCCAAGTGAACAACAATTCATACAACTTAGT 960

PI482399 AAGCATGCATTTGATCAACAAAGTGGGGGTCCAAGTGAACAACAATTCATACAACTTAGT 958

Cornel AAGCATGCATTTGATCAACAAAGTGGGGGTCCAAGTGAACAACAATTCATACAACTTAGT 960

************************************************************

MELO3C022157T1 CAGAATATAGTGGAAAAGGTCGAAGGAAATCCACAAGCATTGGAACAAATTGGATCATTT 1020

PI482399 CAGAATATAGTGGAAAAGGTCGAAGGAAATCCACAAGCATTGGAACAAATTGGATCATTT 1018

Cornel CAGAATATAGTGGAAAAGGTCGAAGGAAATCCACAAGCATTGGAACAAATTGGATCATTT 1020

************************************************************

MELO3C022157T1 TTGCGTGGTAAAGATATTAATGTATGGAAAGAAGAATTGAAGAGCCTTGTTTTAGTTGAT 1080

PI482399 TTGCGTGGTAAAGATATTAATGTATGGAAAGAAGAATTGAAGAGCCTTGTTTTAGTTGAT 1078

Cornel TTGCGTGGTAAAGATATTAATGTATGGAAAGAAGAATTGAAGAGCCTTGTTTTAGTTGAT 1080

************************************************************

MELO3C022157T1 AATGAACGTCTCTTCAAAATATTAAAGATAAGTTTTGATCAATTAGGGACAAAAGGCCAA 1140

PI482399 AATGAACGTCTCTTCAAAATATTAAAGATAAGTTTTGATCAATTAGGGACAAAAGGCCAA 1138

Cornel AATGAACGTCTCTTCAAAATATTAAAGATAAGTTTTGATCAATTAGGGACAAAAGGCCAA 1140

************************************************************

MELO3C022157T1 CAAGCTTTTCTTGATTTGGCATGTTTCTTCAATGGAAAAAGTACAGGCAAAATTATTGAA 1200

PI482399 CAAGCTTTTCTTGATTTGGCATGTTTCTTCAATGGAAAAAGTACAGGCAAAATTATTGAA 1198

Cornel CAAGCTTTTCTTGATTTGGCATGTTTCTTCAATGGAAAAAGTACAGGCAAAATTATTGAA 1200

************************************************************

MELO3C022157T1 ATACTTGCGAGTTTAGAATACAATTCCCCCAGCGAAGTACTAAAGTTGTTGTGTGATAGA 1260

PI482399 ATACTTGCGAGTTTAGAATACAATTCCCCCAGCGAAGTACTAAAGTTGTTGTGTGATAGA 1258

Cornel ATACTTGCGAGTTTAGAATACAATTCCCCCAGCGAAGTACTAAAGTTGTTGTGTGATAGA 1260

************************************************************

MELO3C022157T1 TATCTTATTGAAATTAGAGATGGAGACACAGTATGTATGCCCAATTTGATACAAGAAATG 1320

PI482399 TATCTTATTGAAATTAGAGATGGAGACACAGTATGTATGCCCAATTTGATACAAGAAATG 1318

Cornel TATCTTATTGAAATTAGAGATGGAGACACAGTATGTATGCCCAATTTGATACAAGAAATG 1320

************************************************************

MELO3C022157T1 GGTCGAGAAATAGAACGAAAAAAACGTCAAAGAAGCAGGATTTGGCTTAGAAGAGATGCT 1380

PI482399 GGTCGAGAAATGGACCGACAAGAACGTCAAAGAAGCAGGATTTGGATTAGAAGAGATGCC 1378

Cornel GGTCGAGAAATAGAACGAAAAAAACGTCAAAGAAGCAGGATTTGGCTTAGAAGAGATGCT 1380

***********.**.***.**.***********************.*************

MELO3C022157T1 TTCGACATATTTGATGAACAACATGGAGTAAAAGACATAAAAGGTGTTGTGTTGGACAAG 1440

PI482399 TTCGACATATTTGATGAACAACATGGAGTAAAAGACATAAAAGGTGTTGTGTTGGACAAG 1438

Cornel TTCGACATATCTGATGAACAACATGGAGTAAAAGACATAAAAGGTGTTGTGTTGGACAAG 1440

********** *************************************************

MELO3C022157T1 AGAGACACAGAACCAAACTTAAAGTTGAAGGCTAAACAATTACAAGATATGAGCCGTTTA 1500

PI482399 AGAGACACAGAACCAAACTTAAAGTTGAAGGCTAAACAATTACAAAATATGAGCCGTTTA 1498

Cornel AGAGACACAGAACCAAACTTAAAGTTGAAGGCTAAACAATTACAAGATATGAGCCGTTTA 1500

*********************************************.**************

MELO3C022157T1 AAAATATTAGAGATTGACAATGTGCAGCTGAGTCCAAGAAATCAAAATGATCTCTCAAAT 1560

PI482399 AAAATATTAGAGATTGACAATGTGCAGCTTAGTCCAAGAAATCAAAATGATCTCTCTAAT 1558

Cornel AAAATATTAGAGATTGACAATGTGCAGCTGAGTCCAAGAAATCAAAATGATCTCTCAAAT 1560

***************************** **************************:***

MELO3C022157T1 CAGCTTCGATTGCTCCACTGGGATGGCTTTCCTTCAGACACTTTGCCACTAAATTTCGAA 1620

PI482399 CAGCTTCGATTGCTTCACTGGGATGGCTTTCCTTCAGACACTTTGCCACTAAATTTTGAA 1618

Cornel CAGCTTCGATTGCTCCACTGGGATGGCTTTCCTTCAGACACTTTGCCACTAAATTTCGAA 1620

************** ***************************************** ***

MELO3C022157T1 GCACCATATTTATTTGAACTTCTCTTGCCTAATGCACAAACCACTCATCTTTGGAAAGAA 1680

PI482399 GCACCATATTTATTTGAACTTCTCTTGCCTAATGCTCAAACCACTCATCTTTGGAAAGAA 1678

Cornel GCACCATATTTATTTGAACTTCTCTTGCCTAATGCACAAACCACTCATCTTTGGAAAGAA 1680

***********************************:************************

MELO3C022157T1 CTAAAGGGATTTAAGAAATTAAAGGTAATCGATGTTAGCAATTCCCAAACTTTGGTGGAG 1740

PI482399 CTAAAGGGATTTAAGAAATTAAAGGTAATCGATGTTAGCAATTCCCGAACTTTGGTGGAG 1738

Cornel CTAAAGGGATTTAAGAAATTAAAGGTAATCGATGTTAGCAATTCCCAAACTTTGGTGGAG 1740

**********************************************.*************

MELO3C022157T1 ACACCGAATTTGAGTGCTGTTCCAAATCTAGAAAGATTGATTCTATGTAATTGTACAAGA 1800

PI482399 ACACCGAATTTAAGTGCTGTTCCAAATCTAGAAAGATTGATTCTATGTAATTGTACAAGA 1798

Cornel ACACCGAATTTGAGTGCTGTTCCAAATCTAGAAAGATTGATTCTATGTAATTGTACAAGA 1800

***********.************************************************

MELO3C022157T1 TTGAAGAAAATTGATAATTCAATTACAAAATTGAGACTTCTAGTTTTAGTAGACCTCACA 1860

PI482399 TTGAAGAAAATTGATAATTCAATTACAAAATTGAGACTTCTAGTTTTAGTAGACCTCACA 1858

Cornel TTGAAGAAAATTGATAATTCAATTACAAAATTGAGACTTCTAGTTTTAGTAGACCTCACA 1860

************************************************************

MELO3C022157T1 GGCTGTGTTCGCCTCGAAACATCCGAGTGCATCGATATCTTGAAGAGTCGCCCAACAGTA 1920

PI482399 GGCTGTGTTCGCCTCGAAACATCCGAGTGCATCGATATCTTAAAGAGTCGCCCAACAGTA 1918

Cornel GGCTGTGTTCGCCTCGAAACATCCGAGTGCATCGATATCTTGAAGAGTCGCCCAACAGTA 1920

*****************************************.******************

MELO3C022157T1 GAACTTCGTGGCTTAGTTCTACAGTGTCGCCAATC-------ATTGAAAGGTATTTGTTA 1973

PI482399 GAACTTCGTGGCTTAGTTCTACAGTGTCACCAATCACCAATCATTAGAAGGTATTTGCTA 1978

Cornel GAACTTCGTGGCTTAGTTCTACAGTGTCGCCAATC-------ATTGAAAGGTATTTGTTA 1973

****************************.****** ***..********** **

**7-bp insertion (4^th^ exon)**

MELO3C022157T1 TATTCGTAAATTTTTTCACTTCTTTTTTAAAATTCAGGATCATTTTGTGTAA 2025

PI482399 TATTTGTAAATTCTTTCGCTTATCTTTTAAAATTTAGGATCATCTCGTGTAA 2030

Cornel TATTCGTAAATTTTTTCACTTCTTTTTTAAAATTCAGGATCATTTTGTGTAA 2025

**** ******* ****.***.* ********** ******** * ******

**Figure S4.** Sequence information obtained by cloning and sequencing of melon resistant (PI482399) and susceptible (Cornell ZPPM 339) lines for a TIR-NBS-LRR gene (MELO3C022157) and their alignments. A, Genomic sequence and their alignment; B, ORF prediction of cloned sequences using ORF Finder in NCBI (<https://www.ncbi.nlm.nih.gov/orffinder/>); C, their sequence alignments along with the references sequences retrieved from Cucurbits Genomics Database (<http://cucurbitgenomics.org>) considering ‘DHL92’ as reference genome database. In sequence alignment asterisks (*) indicate sequence similarity. Absence of asterisks indicates sequence dissimilarity. Endesh (-) indicates insertion/deletion of nucleotides. The grey highlights in the genomic DNA sequences show the position of exons. The red and green highlights in the alignment of genomic DNA sequences indicate the GSB9-kh-1-F and GSB9-kh-1-R, respectively. The red and green text colors in the alignment of genomic sequences indicate the GSB9-kh-2-F and GSB9-kh-2-R, respectively.
