# Supplementary material for: Gummy Stem Blight Resistance in Melon: Inheritance Pattern and Development of Molecular Markers
Source: Int J Mol Sci. 2018 Sep 25;19(10):2914. doi: 10.3390/ijms19102914 (PMC6213961; doi:10.3390/ijms19102914)
Supplement: Supplementary file 1 [file ijms-19-02914-s001.zip › Supplementary data/Figure S5.pptx]

## Slide 1
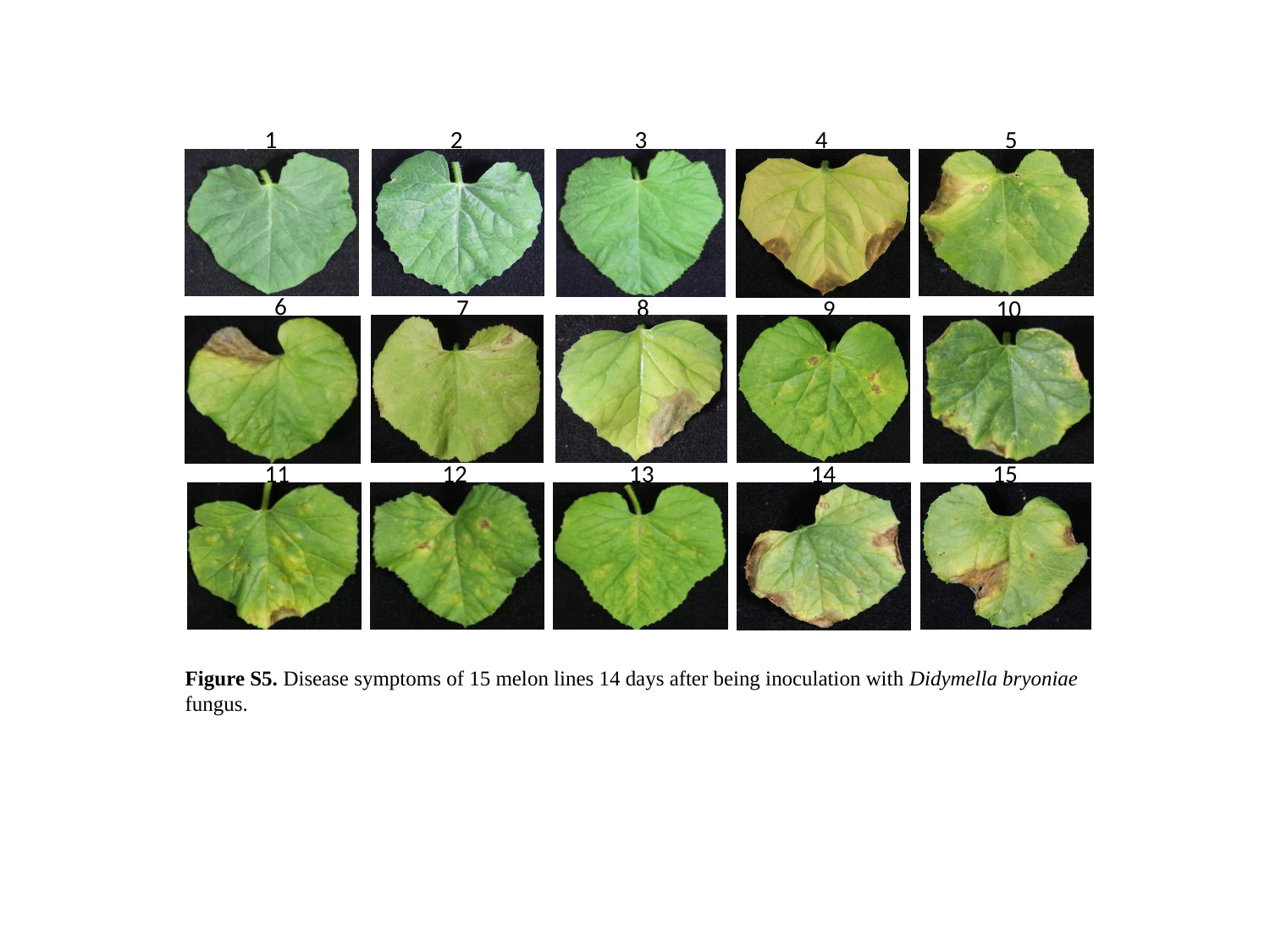

3
4
5
2
1
6
8
7
9
10
13
11
12
15
14
Figure S5. Disease symptoms of 15 melon lines 14 days after being inoculation with Didymella bryoniae
fungus.
