# Supplementary material for: Gummy Stem Blight Resistance in Melon: Inheritance Pattern and Development of Molecular Markers
Source: Int J Mol Sci. 2018 Sep 25;19(10):2914. doi: 10.3390/ijms19102914 (PMC6213961; doi:10.3390/ijms19102914)
Supplement: Supplementary file 1 [file ijms-19-02914-s001.zip › Supplementary data/Table S10.docx]

**Table S10.** Primer specifications of melon resistance genes designed from chromosome 1 and Gummy stem blight resistance genes (*Gsb*) from chromosome 4.

| Gene ID | Chromosome. No. | Forward Primer | Product  Size |
| --- | --- | --- | --- |
| MELO3C013195 | Chr1 | F: TCCCTTTCTTCCTCCTCATT  R: AATTTGGAAAAGGTCGAAGG | 1506 |
| MELO3C012515 | Chr1 | F: TGGCTTTAACCTCCCACAC  R: CAGCACCAGTAACGTTCAAA | 1921 |
| MELO3C012986 | Chr4 | F: GCCAAAGATCCTCCAATTCT  R: CAACCAATGGGGTACTTGAC | 1129 |
| MELO3C012987 | Chr4 | F: CCCTTTAGAAATCCCTCCTC  R: TCGTCAATTTTCAACCCATC | 823 |
| MELO3C012988 | Chr4 | F: TGGATTACCTTAGCAGGCTCT  R: GAGCGATAGTAGTTGCTTCTGG | 901 |
| MELO3C012989 | Chr4 | F: TGGATAGATGGTGGGATCTT  R: CAAGAAGGGGGCACAAAG | 455 |
| MELO3C012990 | Chr4 | F: TGGTCAGGTTTGAAAAATGG  R: CCATCCCCCTTTGGATCT | 1358 |
| MELO3C012991 | Chr4 | F: GCACGAACTTGGAACTCAAT  R: GCACCTGTGACAACAAACAA | 1227 |
| MELO3C012992 | Chr4 | F: ATGGAACTCCCCGAGTATCT  R: ACTAAAATCACCGCCGTTGT | 960 |
| MELO3C012993 | Chr4 | F: CGTGACTCTCGTTTCGTTTT  R: CATTGGGAATTCCTTCACAC | 1398 |
