# Supplementary material for: Gummy Stem Blight Resistance in Melon: Inheritance Pattern and Development of Molecular Markers
Source: Int J Mol Sci. 2018 Sep 25;19(10):2914. doi: 10.3390/ijms19102914 (PMC6213961; doi:10.3390/ijms19102914)
Supplement: Supplementary file 1 [file ijms-19-02914-s001.zip › Supplementary data/Table S2.docx]

**Table S2.** Primer specifications in four locations of candidate GSB resistance NBS-LRR gene MELO3C022157

| Gene ID and Locations | Forward sequence | Reverse sequence | Product size |
| --- | --- | --- | --- |
| MELO3C022157-P1 (1^st^ Exon) | ATGGAAGCAATTGAGGAATC | CTGTTTTTGTTTACTACGACCC | 302 |
| MELO3C022157-P2 (First Intron) | GTAGGGTCTCACTCTTCTCA | CTGCATGGCCAAAGAGTAGT | 876 |
| MELO3C022157-P3 (Second Exon) | CCCTGAAGTGGACAGTATC | TGGGGGAATTGTATTCTAAACTCG | 929 |
| MELO3C022157-P4 (Second Exon-4^th^ Exon) | GCGAAGTACTAAAGTTGTTGTG | TCGGGAGCGAGGGTCATGGA | 1007 |
