# Supplementary material for: Gummy Stem Blight Resistance in Melon: Inheritance Pattern and Development of Molecular Markers
Source: Int J Mol Sci. 2018 Sep 25;19(10):2914. doi: 10.3390/ijms19102914 (PMC6213961; doi:10.3390/ijms19102914)
Supplement: Supplementary file 1 [file ijms-19-02914-s001.zip › Supplementary data/Table S3.docx]

**Table S3**. Interaction between bioassay results with *Didymella. bryoniae* isolate (12-003) and InDel marker (GSB9-kh-1, GSB9-kh-2) bands against GSB resistant gene for melon F_2_ population. R, resistant and S, susceptible indicate status of interactions between each melon F_2_ plant and fungal isolate. Plus (+) sign favours resistant polymorphic marker type and minus (–) sign indicates absence of resistant marker type.

| Melon F_2_ Population | Percent Disease Index (PDI) | Phenotype | Genotype | |
| --- | --- | --- | --- | --- |
|  |  |  | InDel marker  (GSB9-kh-1) | InDel marker  (GSB9-kh-2) |
| 1 | 64 | S | **–** | **–** |
| 2 | 20 | R | **+** | **+** |
| 3 | 20 | R | **+** | **+** |
| 4 | 70 | S | **–** | **–** |
| 5 | 68 | S | **–** | **–** |
| 6 | 60 | S | **–** | **–** |
| 7 | 76 | S | **–** | **–** |
| 8 | 60 | S | **–** | **–** |
| 9 | 68 | S | **–** | **–** |
| 10 | 80 | S | **–** | **–** |
| 11 | 60 | S | **–** | **–** |
| 12 | 62 | S | **–** | **–** |
| 13 | 70 | S | **–** | **–** |
| 14 | 20 | R | **+** | **+** |
| 15 | 90 | S | **–** | **–** |
| 16 | 58 | S | **–** | **–** |
| 17 | 62 | S | **–** | **–** |
| 18 | 70 | S | **–** | **–** |
| 19 | 20 | R | **+** | **+** |
| 20 | 20 | R | **+** | **+** |
| 21 | 20 | R | **+** | **+** |
| 22 | 72 | S | **–** | **–** |
| 23 | 20 | R | **+** | **+** |
| 24 | 20 | R | **+** | **+** |
| 25 | 74 | S | **–** | **–** |
| 26 | 68 | S | **–** | **–** |
| 27 | 20 | R | **+** | **+** |
| 28 | 74 | S | **–** | **–** |
| 29 | 76 | S | **–** | **–** |
| 30 | 58 | S | **–** | **–** |
| 31 | 60 | S | **–** | **–** |
| 32 | 74 | S | **–** | **–** |
| 33 | 60 | S | **–** | **–** |
| 34 | 88 | S | **–** | **–** |
| 35 | 80 | S | **–** | **–** |
| 36 | 78 | S | **–** | **–** |
| 37 | 78 | S | **–** | **–** |
| 38 | 20 | R | **+** | **+** |
| 39 | 78 | S | **–** | **–** |
| 40 | 80 | S | **–** | **–** |
| 41 | 60 | S | **–** | **–** |
| 42 | 70 | S | **–** | **–** |
| 43 | 58 | S | **–** | **–** |
| 44 | 20 | R | **+** | **+** |
| 45 | 20 | R | **+** | **+** |
| 46 | 60 | S | **–** | **–** |
| 47 | 64 | S | **–** | **–** |
| 48 | 20 | R | **+** | **+** |
| 49 | 80 | S | **–** | **–** |
| 50 | 74 | S | **–** | **–** |
| 51 | 66 | S | **–** | **–** |
| 52 | 74 | S | **–** | **–** |
| 53 | 76 | S | **–** | **–** |
| 54 | 20 | R | **–** | **–** |
| 55 | 20 | R | **+** | **+** |
| 56 | 72 | S | **–** | **–** |
| 57 | 20 | R | **+** | **+** |
| 58 | 58 | S | **–** | **–** |
| 59 | 56 | S | **–** | **–** |
| 60 | 20 | R | **+** | **+** |
| 61 | 64 | S | **–** | **–** |
| 62 | 58 | S | **–** | **–** |
| 63 | 20 | R | **+** | **+** |
| 64 | 70 | S | **–** | **–** |
| 65 | 74 | S | **–** | **–** |
| 66 | 72 | S | **–** | **–** |
| 67 | 78 | S | **–** | **–** |
| 68 | 20 | R | **+** | **+** |
| 69 | 56 | S | **–** | **–** |
| 70 | 64 | S | **–** | **–** |
| 71 | 78 | S | **–** | **–** |
| 72 | 80 | S | **–** | **–** |
| 73 | 82 | S | **–** | **–** |
| 74 | 58 | S | **–** | **–** |
| 75 | 70 | S | **–** | **–** |
| 76 | 72 | S | **–** | **–** |
| 77 | 80 | S | **–** | **–** |
| 78 | 64 | S | **–** | **–** |
| 79 | 58 | S | **–** | **–** |
| 80 | 70 | S | **–** | **–** |
| 81 | 58 | S | **–** | **–** |
| 82 | 54 | S | **–** | **–** |
| 83 | 84 | S | **–** | **–** |
| 84 | 60 | S | **–** | **–** |
| 85 | 70 | S | **–** | **–** |
| 86 | 86 | S | **–** | **–** |
| 87 | 68 | S | **–** | **–** |
| 88 | 64 | S | **–** | **–** |
| 89 | 58 | S | **–** | **–** |
| 90 | 88 | S | **–** | **–** |
| 91 | 62 | S | **–** | **–** |
| 92 | 76 | S | **–** | **–** |
| 93 | 60 | S | **–** | **–** |
| 94 | 20 | R | **+** | **+** |
| 95 | 76 | S | **–** | **–** |
| 96 | 46 | S | **–** | **–** |
| 97 | 60 | S | **–** | **–** |
| 98 | 86 | S | **–** | **–** |
| 99 | 64 | S | **–** | **–** |
| 100 | 56 | S | **–** | **–** |
| 101 | 84 | S | **–** | **–** |
| 102 | 68 | S | **–** | **–** |
| 103 | 58 | S | **–** | **–** |
| 104 | 70 | S | **–** | **–** |
| 105 | 60 | S | **–** | **–** |
| 106 | 58 | S | **–** | **–** |
| 107 | 20 | R | **+** | **+** |
| 108 | 20 | R | **+** | **+** |
| 109 | 60 | S | **–** | **–** |
| 110 | 20 | R | **+** | **+** |
| 111 | 64 | S | **–** | **–** |
| 112 | 68 | S | **–** | **–** |
| 113 | 68 | S | **–** | **–** |
| 114 | 20 | R | **+** | **+** |
| 115 | 60 | S | **–** | **–** |
| 116 | 20 | R | **+** | **+** |
| 117 | 20 | R | **+** | **+** |
| 118 | 64 | S | **–** | **–** |
| 119 | 70 | S | **–** | **–** |
| 120 | 20 | R | **+** | **+** |
| 121 | 60 | S | **–** | **–** |
| 122 | 68 | S | **–** | **–** |
| 123 | 74 | S | **–** | **–** |
| 124 | 60 | S | **–** | **–** |
| 125 | 76 | S | **–** | **–** |
| 126 | 20 | R | **+** | **+** |
| 127 | 70 | S | **–** | **–** |
| 128 | 20 | R | **+** | **+** |
| 129 | 20 | R | **+** | **+** |
| 130 | 58 | S | **–** | **–** |
| 131 | 70 | S | **–** | **–** |
| 132 | 56 | S | **–** | **–** |
| 133 | 60 | S | **–** | **–** |
| 134 | 20 | R | **+** | **+** |
| 135 | 74 | S | **–** | **–** |
| 136 | 84 | S | **–** | **–** |
| 137 | 20 | R | **+** | **+** |
| 138 | 66 | S | **–** | **–** |
| 139 | 70 | S | **–** | **–** |
| 140 | 64 | S | **–** | **–** |
| 141 | 86 | S | **+** | **+** |
| 142 | 20 | R | **+** | **+** |
| 143 | 52 | S | **–** | **–** |
| 144 | 58 | S | **–** | **–** |
| 145 | 60 | S | **–** | **–** |
| 146 | 60 | S | **–** | **–** |
| 147 | 74 | S | **–** | **–** |
| 148 | 58 | S | **–** | **–** |
| 149 | 70 | S | **–** | **–** |
| 150 | 20 | R | **+** | **+** |
| 151 | 20 | R | **+** | **+** |
| 152 | 60 | S | **–** | **–** |
| 153 | 60 | S | **–** | **–** |
| 154 | 20 | R | **+** | **+** |
| 155 | 20 | R | **+** | **+** |
| 156 | 58 | S | **–** | **–** |
| 157 | 20 | R | **+** | **+** |
| 158 | 60 | S | **–** | **–** |
| 159 | 66 | S | **–** | **–** |
| 160 | 20 | R | **+** | **+** |
| 161 | 20 | R | **+** | **+** |
| 162 | 60 | S | **–** | **–** |
| 163 | 72 | S | **–** | **–** |
| 164 | 58 | S | **–** | **–** |
| 165 | 20 | R | **+** | **+** |
| 166 | 60 | S | **–** | **–** |
| 167 | 78 | S | **–** | **–** |
| 168 | 20 | R | **+** | **+** |
