# Supplementary material for: Gummy Stem Blight Resistance in Melon: Inheritance Pattern and Development of Molecular Markers
Source: Int J Mol Sci. 2018 Sep 25;19(10):2914. doi: 10.3390/ijms19102914 (PMC6213961; doi:10.3390/ijms19102914)
Supplement: Supplementary file 1 [file ijms-19-02914-s001.zip › Supplementary data/Table S4.docx]

Table S4. Interaction between bioassay results with *Didymella. bryoniae* isolate (12-003) and InDel marker (GSB9-kh-1, GSB9-kh-2) bands against Gsb resistant gene for melon 15 lines. R, resistant and S, susceptible indicate status of interactions between each melon line and fungal isolate. Plus (+) sign favours resistant polymorphic marker type and minus (–) sign indicates absence of resistant marker type.

| Sl No. | Melon lines | Percent Disease Index (PDI) | Phenotype | Genotype | |
| --- | --- | --- | --- | --- | --- |
|  |  |  |  | InDel marker (GSB9-kh-1) | InDel marker (GSB9-kh-2) |
| 1 | PI482398 | 20 | R | **+** | **+** |
| 2 | PI353814 | 20 | R | **+** | **+** |
| 3 | PI504558 | 20 | R | **+** | **+** |
| 4 | PI157076 | 60 | S | **–** | **–** |
| 5 | PI157082 | 72 | S | **–** | **–** |
| 6 | PI614601 | 70 | S | **–** | **–** |
| 7 | PI614525 | 74 | S | **–** | **–** |
| 8 | PI536473 | 70 | S | **–** | **–** |
| 9 | SCNU1154 | 72 | S | **–** | **–** |
| 10 | Honeydew Greenflesh | 70 | S | **–** | **–** |
| 11 | MR1 | 88 | S | **–** | **–** |
| 12 | PMR5 | 76 | S | **–** | **–** |
| 13 | PMR45 | 74 | S | **–** | **–** |
| 14 | WMR29 | 74 | S | **–** | **–** |
| 15 | Edisto47 | 72 | S | **–** | **–** |
