# Supplementary material for: Gummy Stem Blight Resistance in Melon: Inheritance Pattern and Development of Molecular Markers
Source: Int J Mol Sci. 2018 Sep 25;19(10):2914. doi: 10.3390/ijms19102914 (PMC6213961; doi:10.3390/ijms19102914)
Supplement: Supplementary file 1 [file ijms-19-02914-s001.zip › Supplementary data/Table S5.docx]

**Table S5.** Features of NBS-LRR gene MELO3C022157

| Homology | Match Name | E-value | % Identity | Description |
| --- | --- | --- | --- | --- |
| [BLAST of MELO3C022157 vs. NCBI nr](http://cucurbitgenomics.org/feature/gene/MELO3C022157) | [gi\|659119254\|ref\|XP_008459557.1\|](http://www.ncbi.nlm.nih.gov/protein/659119254) | 0.0e+00 | 99.85 | PREDICTED: TMV resistance protein N-like isoform X3 [*Cucumis melo*] |
| [BLAST of MELO3C022157 vs. TAIR10](http://cucurbitgenomics.org/feature/gene/MELO3C022157) | [AT1G17600.1](http://www.arabidopsis.org/servlets/TairObject?type=locus&name=AT1G17600) | 4.0e-85 | 35.85 | Disease resistance protein (TIR-NBS-LRR class) family |
| [BLAST of MELO3C022157 vs. TrEMBL](http://cucurbitgenomics.org/feature/gene/MELO3C022157) | [M4QSV9_CUCME](http://www.uniprot.org/uniprot/M4QSV9) | 1.7e-161 | 50.15 | Fom-1 OS=*Cucumis melo* GN=Fom-1 PE=4 SV=1 |
| [BLAST of MELO3C022157 vs. Swiss-Prot](http://cucurbitgenomics.org/feature/gene/MELO3C022157) | [TMVRN_NICGU](http://www.uniprot.org/uniprot/Q40392) | 1.2e-91 | 34.01 | TMV resistance protein N OS=*Nicotiana glutinosa* GN=N PE=1 SV=1 |
