# Supplementary material for: Gummy Stem Blight Resistance in Melon: Inheritance Pattern and Development of Molecular Markers
Source: Int J Mol Sci. 2018 Sep 25;19(10):2914. doi: 10.3390/ijms19102914 (PMC6213961; doi:10.3390/ijms19102914)
Supplement: Supplementary file 1 [file ijms-19-02914-s001.zip › Supplementary data/Table S6.docx]

**Table S6.** Primer specifications of SSR marker CMCT505 linked to gummy stem blight resistance in melon chromosome 1

| Marker Name | Forward primer | Reverse primer | Product size (bp) |
| --- | --- | --- | --- |
| SSR (CMCT505) | GACAGTAATCACCTCATCAAC | GGGAATGTAAATTGGATATG | 210 |
