# Supplementary material for: Gummy Stem Blight Resistance in Melon: Inheritance Pattern and Development of Molecular Markers
Source: Int J Mol Sci. 2018 Sep 25;19(10):2914. doi: 10.3390/ijms19102914 (PMC6213961; doi:10.3390/ijms19102914)
Supplement: Supplementary file 1 [file ijms-19-02914-s001.zip › Supplementary data/Table S8.docx]

**Table S8**. List of melon lines used for validation of InDel markers (Gsb9-kh-1 and Gsb9-kh-2) resistance to *Didymella bryoniae*

| Sl. No. | Genotype | Seed Source |
| --- | --- | --- |
| 1 | PI482398 | CU, USDA |
| 2 | PI353814 | CU, USA |
| 3 | PI504558 | USDA, USA |
| 4 | PI157076 | USDA, USA |
| 5 | PI157082 | USDA, USA |
| 6 | PI614601 | USDA, USA |
| 7 | PI614525 | USDA, USA |
| 8 | PI536473 | USDA, USA |
| 9 | SCNU1154 | SCNU, Korea |
| 10 | Honeydew Greenflesh | CU, USA |
| 11 | MR1 | USDA, USA |
| 12 | PMR5 | USDA, USA |
| 13 | PMR45 | USDA, USA |
| 14 | WMR29 | USDA, USA |
| 15 | Edisto47 | USDA, USA |

CU-Cornell University, United States of America and USDA-United States Department of Agriculture, SCNU- Sunchon National Unversity, South Korea
