# Supplementary material for: Gummy Stem Blight Resistance in Melon: Inheritance Pattern and Development of Molecular Markers
Source: Int J Mol Sci. 2018 Sep 25;19(10):2914. doi: 10.3390/ijms19102914 (PMC6213961; doi:10.3390/ijms19102914)
Supplement: Supplementary file 1 [file ijms-19-02914-s001.zip › Supplementary data/Table S9.docx]

**Table S9.** Percent disease index (PDI) of resistant and susceptible lines and their F_1_ crosses.

| Melon lines and F_1_ | Generation | Percent Disease Index (PDI) |
| --- | --- | --- |
| PI482399 (Resistant) | P1 | 20 |
| Cornell ZPPM 339 (Susceptible) | P2 | 94 |
| Cornell × PI482399 | F_1_ | 90 |
