# Supplementary figures and images for: Gummy Stem Blight Resistance in Melon: Inheritance Pattern and Development of Molecular Markers
Source: Int J Mol Sci. 2018 Sep 25;19(10):2914. doi: 10.3390/ijms19102914 (PMC6213961; doi:10.3390/ijms19102914)

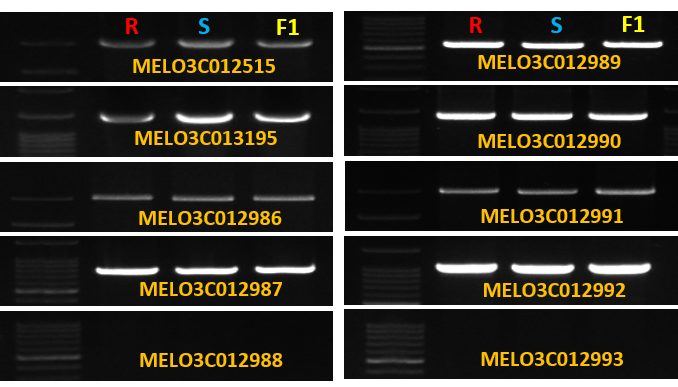

Supplement: Supplementary file 1 [file ijms-19-02914-s001.zip › Supplementary data/Figure S2.tif]

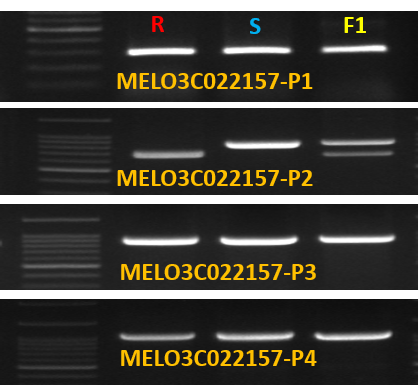

Supplement: Supplementary file 1 [file ijms-19-02914-s001.zip › Supplementary data/Figure S3.tif]

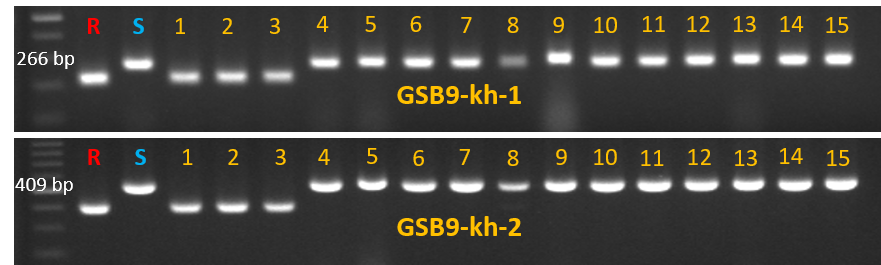

Supplement: Supplementary file 1 [file ijms-19-02914-s001.zip › Supplementary data/Figure S6.tif]

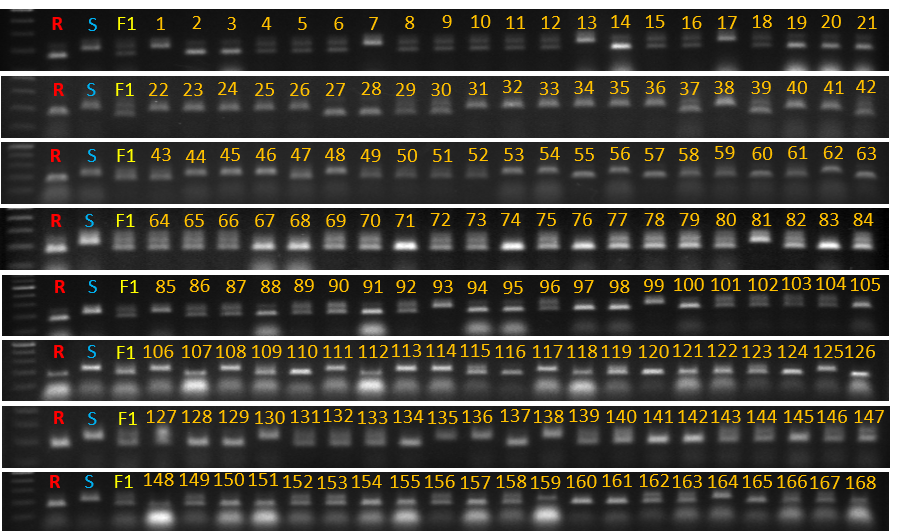

Supplement: Supplementary file 1 [file ijms-19-02914-s001.zip › Supplementary data/Figure S7.tif]

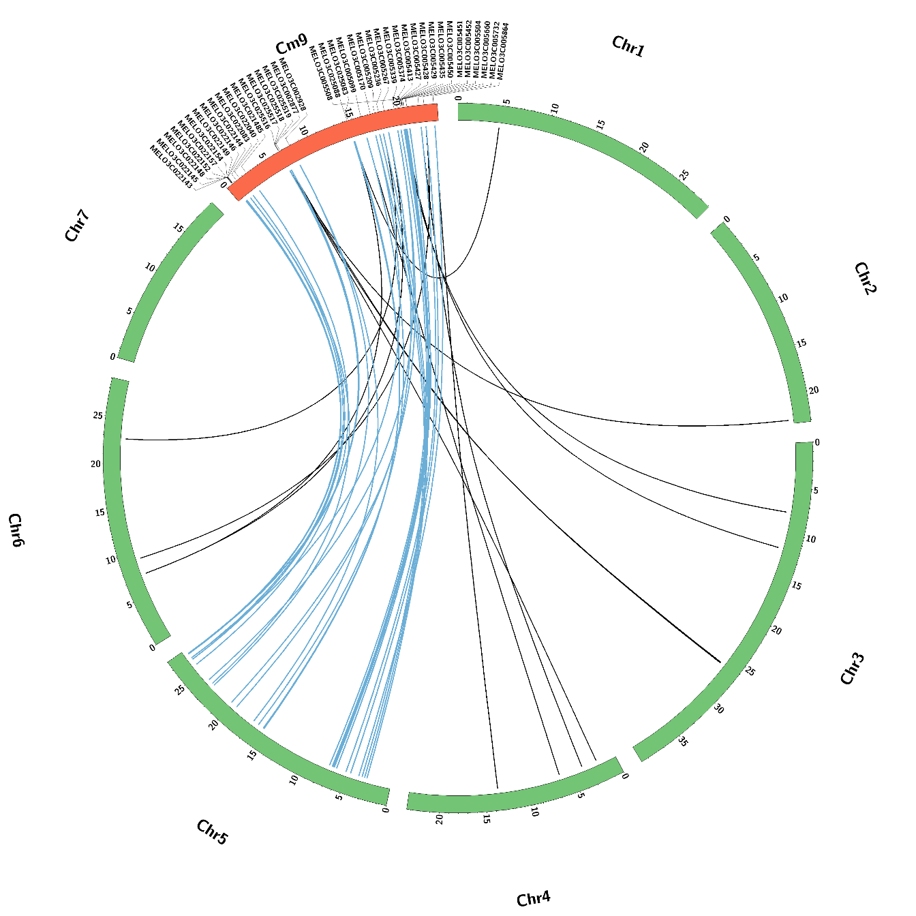

Supplement: Supplementary file 1 [file ijms-19-02914-s001.zip › Supplementary data/Figure S8.tif]
